# Supplementary material for: Wireless, battery-free, multifunctional integrated bioelectronics for respiratory pathogens monitoring and severity evaluation
Source: Nat Commun. 2023 Nov 20;14:7539. doi: 10.1038/s41467-023-43189-z (PMC10661182; doi:10.1038/s41467-023-43189-z)
Supplement: Supplementary file 1 — Supplementary Information [file 41467_2023_43189_MOESM1_ESM.pdf]

Supporting Information for

Wireless, battery-free, multifunctional integrated bioelectronics for respiratory pathogens monitoring and severity evaluation

Hu Li,[1,2,11] Huarui Gong,[3,4,11] Tsz Hung Wong,[1,11] Jingkun Zhou,[1,5,11] Yuqiong Wang,[2,11] Long Lin,[6] Ying Dou,[4] Huiling Jia,[1,5] Xingcan Huang,[1] Zhan Gao,[1] Rui Shi,[1] Ya Huang,[1,5] Zhenlin Chen,[1] Wooyoung PARK,[1] Ji Yu Li,[1,5] Hongwei Chu,[1] Shengxin Jia,[1] Han Wu,[2] Mengge Wu,[1] Yiming Liu,[1] Dengfeng Li,[1] Jian Li,[1] Guoqiang Xu,[1] Tianrui Chang,[2] Binbin Zhang,[1,5] Yuyu Gao,[1] Jingyou Su,[1] Hao Bai,[7] Jie Hu,[7] Chun Ki Yiu,[1,5] Chenjie Xu,[1] Wenchuang Hu,[7*] Jiandong Huang,[3,4,8,9*] Lingqian Chang,[2,10*] Xinge Yu[1,5*]

1 Department of Biomedical Engineering, City University of Hong Kong, Hong Kong 999077, China

2 Beijing Advanced Innovation Center for Biomedical Engineering, School of Biological Science and Medical Engineering, Beihang University, Beijing 100083, China.

3 Key Laboratory of Quantitative Synthetic Biology, Shenzhen Institute of Synthetic Biology, Shenzhen Institutes of Advanced Technology, Chinese Academy of Sciences, Shenzhen 518055, China

4 School of Biomedical Sciences, Li Ka Shing Faculty of Medicine, University of Hong Kong, Hong Kong 999077, China

5 Hong Kong Centre for Cerebro-Cardiovascular Health Engineering, Hong Kong 999077, China

6 College of Engineering, Peking University, Beijing, 100871, China

7 Department of Laboratory Medicine, Med+X Center for Manufacturing, West China Precision Medicine Industrial Technology Institute, Department of Liver Surgery, Department of Pathology, West China Hospital, Sichuan University, Chengdu, Sichuan, 610041, China

8 Clinical Oncology Center, Shenzhen Key Laboratory for cancer metastasis and personalized therapy, The University of Hong Kong-Shenzhen Hospital, Shenzhen, China

9 Guangdong-Hong Kong Joint Laboratory for RNA Medicine, Sun Yat-Sen University, Guangzhou, China 510120.

10 School of Biomedical Engineering, Research and Engineering Center of Biomedical Materials, Anhui Medical University, Hefei 230032, China

11 These authors contributed equally to this work.

*Email: xingeyu@cityu.edu.hk; lingqianchang@buaa.edu.cn; jdhuang@hku.hk; huwenchuang@wchscu.cn

List of Supplementary Contents

Supplementary Table 1 SARS-CoV-2 detection by saliva, blood and nasopharyngeal secretions………………………………………………………………………....5

Supplementary Table 2 Breath related works for SARS-CoV-2 detection.................6

Supplementary note 1

Innovations and significance by comparing our work with the pioneering works in Supplementary Tables 1 and 2……………………….………………….……..…....7-10

Supplementary Table 3 Information of 21 negative cases..11

Supplementary Table 4 Information of 21 positive cases..12

List of Supplementary Figures

Fig. 1 Outlook of pandemic screening and diagnosis by breath and blow................13

Fig. 2 Pictures of the packaged PIDS in different states...14

Fig. 3 Preparation process of the multifunctional PIDS for monitoring the exhaled virus, respiration rate and exhaled breath temperature..15

Fig. 4 Preparation process of the circular PIDS...16

Fig. 5 The hydrophobicity of PDMS contributed to keeping the air-liquid interface stable in different directions..17

Fig. 6 Simulation of the air-liquid interface pressure with different inlet numbers in the symmetrical stable condition…………………...18

Fig. 7 Simulation of airflow velocity at the outlet end with different inlet numbers in the symmetrical stable condition…………………………….............................19

Fig. 8 Simulation of volume fraction of PBS with different airflow velocities in the asymmetrical transient condition……………………………………………………20

Fig. 9 Simulation of the air-liquid interface pressure with different airflow velocities in the asymmetrical transient condition………………………….……………..….…..21

Fig. 10 Liquid change in chamber with different strength of blow……………...……22

Fig. 11 Simulation of the air-liquid interface pressure with different outlet lengths in

the symmetrical stable condition……………………..23
Fig. 12 Simulation of airflow velocity at the outlet end with different outlet lengths in the symmetrical stable condition…………………………….........................24
Fig. 13 Simulation of the air-liquid interface pressure with different outlet widths in the symmetrical stable condition……………………………………………….....25
Fig. 14 Simulation of airflow velocity at the outlet end with different outlet widths in the symmetrical stable condition…………………………………......................26
Fig. 15 Simulation of the rapid collection of breath sample and blow with an atomizer..27
Fig. 16 Collection simulation of aerosols using an atomizer………………………28
Fig. 17 Collection speed of the aerosols with mouth blow………………………..29
Fig. 18 Surface morphologies and characteristic elements of the activated graphene...30
Fig. 19 Current change after modifying PBASE and the spike antibody....................31
Fig. 20 Limit of detection (LOD), specific identification and selective identification of IBS module to SARS-CoV-2 spike proteins by using different antibodies................32
Fig. 21 Detection performance of the IBS module to SARS-CoV-2 pseudovirus including WT, Alpha, Delta and Omicron...33
Fig. 22 Detection performance of the IBS module to the live viruses including WT, Alpha, Delta and Omicron...34
Fig. 23 Ages of the 21 negative participants..35
Fig. 24 Data of the positive case 1 to case 10..36
Fig. 25 Data of the positive case 11 to case 20...37
Fig. 26 Data of the positive case 21...38
Fig. 27 Data of the negative case 1 to case 10...39
Fig. 28 Data of the negative case 11 to case 20...40
Fig. 29 Data of the negative case 21………...41
Fig. 30 Respiratory characteristics recorded by the RBS module............................42
Fig. 31 Respiratory rate (RR) calculation of a regular breath curve…………….....43
Fig. 32 Respiratory rate (RR) calculation of an irregular breath curve………………44
Fig. 33 The breath curves were recorded for the negative case 1 to case 5...................45

Fig. 34 The breath curves were recorded for the negative case 6 to case 10.................46
Fig. 35 The breath curves were recorded for the negative case 11 to case 15............ 47
Fig. 36 The breath curves were recorded for the negative case 16 to case 21............ 48
Fig. 37 The breath curves were recorded for the positive case 1 to case 5……………49
Fig. 38 The breath curves were recorded for the positive case 6 to case 10…………50
Fig. 39 The breath curves were recorded for the positive case 11 to case 15…………51
Fig. 40 The breath curves were recorded for the positive case 16 to case 21…………52
Fig. 41 Ambient temperature before and after filtration with the notch filter………..53
Fig. 42 Comparison of accuracy of diagnosing the viral infection without RR and EBT……………………………………………………………………………..54
Fig. 43 Extension of our technology to another two pandemic viruses……………..…55
Fig. 44 Identification ability of IBS to SARS-CoV-2 spike protein under room temperature……………………………………………………………………...56
Fig. 45 Dimension of the interdigital electrodes...57
Fig. 46 The red ink (or PBS solution) was injected into the chamber from the outlet using an injector……………………………………………………………...58
Fig. 47 Pictures of red ink stored at -20 °C…………………………….……….……59
Fig. 48 Real-time current curves recorded by dropwise adding 10 µL of PBS on the IBS module……………………………………………………………………………..60
Fig. 49 Baseline current of IBS module under unknow breath and blow……………61
Fig. 50 Dimension of the bionic coronavirus inspired microchannel.......................62
Fig. 51 Resonance frequency of the NFC circuit..63
Fig. 52 Diagram of the NFC circuit...64
Fig. 53 The verification of differentiating exhaled virus from human activities………65

Supplementary references...66-67

Supplementary Table 1. SARS-CoV-2 detection by saliva, blood and nasopharyngeal secretions

Product form	Time	Power supply	Sample	Identification	Method	Stage	Function	Product picture	Ref.
Portable device	2 hours	external power source	Nasopharyngeal swab	Identify RNA	RNA amplification	Clinical samples	Non-invasive passive screening	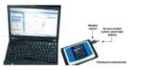	2
Portable device	4 min	Battery-free	Nasopharyngeal swab and saliva	Identify spike protein	Electrochemical impedance spectroscopy	Clinical samples	Non-invasive passive screening	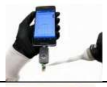	3
Portable device	4 min	Battery	Nasopharyngeal swabs	Identify RNA	Field-effect transistor	Clinical samples	Non-invasive passive screening	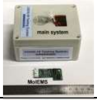	4
Portable device	1 hour	Battery	Saliva	Identify RNA	CRISPR	Clinical samples	Non-invasive passive screening	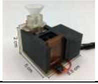	5
Portable device	1 min	Battery	Saliva/Blood	Identify spike proteins	Electrochemical impedance	Clinical samples	Non-invasive passive screening;<br>Invasive passive diagnosis	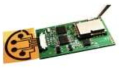	6
Portable device	5 min	Battery	Saliva	Identify IgG	Organic electrochemical transistors	Clinical samples	Non-invasive passive screening	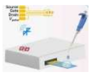	7
Portable device	30 min	Battery	Nasopharyngeal swab	Identify RNA	Isothermal amplification	Clinical samples	Non-invasive passive screening	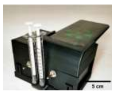	8
Portable device	30 min	Battery	Saliva	Identify RNA	Reverse transcriptase loopmediated isothermal amplification	Clinical samples	Non-invasive passive screening	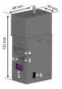	9
Portable equipment	1 hour	External power source	Throat swab/ sputum	Identify RNA	Lateral flow immunoassay	Clinical samples	Non-invasive passive screening	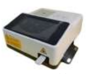 Size: 55.5 cm×37cm×23cm	10
Portable device	20 min	Battery	Saliva	Identify spike protein	Field-effect transistor	Clinical samples	Non-invasive passive screening	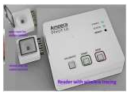	11
Portable equipment	4 min	External power source	Nasopharyngeal swab	Proteomics assays	Mass spectrometry	Clinical samples	Non-invasive passive screening	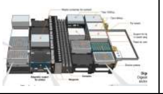	12
Portable device	30 min	Battery-free	Nasopharyngeal swab	Identify RNA	CRISPR-Cas13a	Clinical samples	Non-invasive passive screening	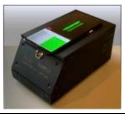	13
Portable device	40 min	External power source	nasopharyngeal swab	Identify RNA	CRISPR-Cas12	Clinical samples	Non-invasive passive screening	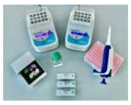	14
Portable device	17 min	Battery	Nasopharyngeal swab/sputum	Identify RNA	Reverse transcription	Clinical samples	Non-invasive passive screening	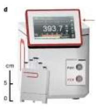	15

Supplementary Table 2. Breath related works for SARS-CoV-2 detection

Product form	Time	Power supply	Sample	Identification	Method	Stage	Function	Product picture	Ref.
Mask	10 min	Battery	Simulated atomizing gas with commercial spike proteins	Identify commercial spike protein	Ion-gated transistor	Commercial proteins in laboratory;	Non-invasive active screening	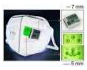	26
Mask	2 hours	Battery-free	Simulated breath with synthetic RNA	Identify synthetic RNA	Lateral flow assay; Fluorescent aptamers; CRISPR-Cas12a	Synthetic RNA fragment in laboratory;	Non-invasive active screening	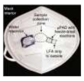	27
Miniaturized, evolvable, multifunctional platform	1 min	Battery-free	Human breath and blow, live virus	Identify spike protein of live SARS-CoV-2 virus; Record respiration rate and exhaled breath temperature	Graphene-based immuno biosensor; Graphene-based breath biosensor; Commercial temperature sensor	Spike protein; Pseudovirus, Live virus; Practical human experiment	Multifunctional breath analysis; Non-invasive active and passive screening and diagnosis of symptom severity	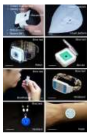	**This work**

Supplementary note 1:

Innovations and significance by comparing our work with the pioneering works in supplementary Tables 1 and 2:

1. **Why choose breath and blow for diagnosing virus infection rather than saliva and swabbed samples?**

The saliva, sputum and swabbed samples dominated the SARS-CoV-2 antigen screening in the past three years because of their easy sampling and high viral loads[1]. Then along comes the rapid development of related point-of-care technologies, especially the portable integrated devices and equipment[2-15]. But on the other hand, the swabbed samplings (i.e., nasopharyngeal and throat swab) have their own limitations as follows.

- First, the swabs usually cause uncomfortable feelings and may not be endured by all patients, particularly infants and the elderly.
- Second, the nonstandard sampling usually misses the areas with high viral loads during the swabbing that resulted in the high false-negative rate with some hurried RT-PCR tests, it even reached 58%[16].
- Third, the trained staffs are required for the swab sampling to reduce the false-negative rate and avoid the inconclusive tests[17, 18].
- Fourth, the samples need the time-consuming transportation for further centralized laboratory assays. The time spent for rural and remote areas could reach 48 hours or longer[19]. The results delivery of PCR needs mostly 1 or 2 days after sampling[20].
- Fifth, the rapid antigenic tests and sensitive molecular tests using swabbed samples have the common limitations in terms of testing procedures, which require the trained personnel and properly equipped test sites. Most of them involves challenges with the operational logistics and product supply chains for the enormous number of tests per day in every country[20].
- Sixth, the sampling operations have huge risks of cross infection between patients and medical workers.

2. **The advantages, challenges, and state of development of breath/blow analysis for virus infection**

Above-mentioned limitations of saliva and swabbed samples may be avoided by analyzing other virus carriers, that is, the breath and blow. From the viral transmission standpoint, respiratory activities showed a much higher priority than swabbed samples. The breath and blow (e.g., speaking, cough and sneeze) can emit thousands of SARS-CoV-2 viruses just within one minute[21-23]. The infected patients can continuously produce viral samples and emit them to environment without need of swab sampling. It suggests that the breath and blow can be alternative solutions for SARS-CoV-2 detection. In 2021, scientists published the perspective about the technical challenges, significance and application potentials of breath analysis, which could be the most appealing approach to consistently monitor COVID-19 spread and a game changer during the pandemic[20]. However, the breath analysis (including blow) of virus infection was underutilized historically even in the COVID-19 pandemic because of the challenging gaseous virus sample collection and onsite antigen identification by platforms themselves, bulky medical equipment (e.g., mass spectrometry analysis), the limited technologies in miniaturized system integration and poor multi-parameter analysis[20, 24, 25]. The pioneering works related to breath using integrated techniques for SARS-CoV-2 detection have yet to be further developed and stayed in the laboratory assay and simulation with commercial proteins and synthetic RNA fragments without human experiments[26, 27].

3. The innovations and contributions of our work that advance the diagnosis of virus infection by breath and blow

Compared with these pioneering works, we made innovations and contributions to the diagnosis of virus infection as follows:

- **The method for rapid sample collection from breath and blow without manual operation.** In our work, we have created a bionic coronavirus-inspired microchannel by which we built the air-liquid interface to continuously dissolve and collect the exhaled viral droplets from breath and blow. Through this simple physical phenomenon, the air-liquid interface realized high efficiency in collecting the gaseous samples from human breath and blow within seconds that meet the requirements for rapid SARS-CoV-2 identification.

- **Onsite identification of SARS-CoV-2 by platform itself.** After collecting the breath and blow samples in the PBS solution in the chamber of microchannel, the antibodies can specifically bind with the virus spike protein and identify the antigen by the immuno biosensor of PIDS, it dispenses with the transportation for further assay using other equipment.
- **Miniaturized system integration.** In our work, we integrated three biosensing modules into an all-in-one miniaturized biosystem (size, 5cm × 5cm × 2 mm) that can be combined with various wearables and transformed into other miniaturized platforms as the handheld breathalyzer, the wearable watch/wristband/necklace, and smart patch for both breath test and blow test. The evolvable property widens the diagnostic modes and broadens its application in multiple life scenarios.
- **Multifunctional monitoring and machine learning deep analysis.** Besides collecting and identifying the exhaled SARS-CoV-2, our system can also monitor the breath rate and breath temperature. The virus signals and physical signs together with the machine learning algorithm enable us to conduct comprehensive diagnosis of virus infection and symptom severity. It may not only help the active and passive screening of populations, but also guide individuals to receive personalized therapy and lower the morbidity of severe infection.
- **Battery-free system**. We adopted the battery-free near-field communication (NFC) technique that wirelessly transmit data just by smartphone touch or walking through the NFC-enabled biosafety doors. This design avoided the frequent replacement of the batteries, dependence of other large power sources and fixed charging positions to ensure the normal operation of the system. It also abandoned the complex connection operations of Bluetooth techniques that limited by password input between different users, especially the public gathering places.
- **Rapid diagnosis speed.** The integrated system in our work can rapidly identify the SARS-CoV-2 viruses from blow within 1 minute and breath within 5 minutes without interrupting the daily life.
- **High diagnosis accuracy.** The system in our work reached to 100% accuracy in identifying the positive and negative cases and 92% accuracy in evaluating their

symptom severity.

- **Simple operation steps.** The only step the users need to do is the smartphone touch without the need of any professional training.
- **Relative mature stage.** We systematically carried out the experiment by verification of spike proteins, pseudo virus, live virus and practical human diagnosis beyond the laboratory assay.

4. The potential significance of our work

Compared with the techniques based on saliva, blood and nasopharyngeal secretions, the breath- and blow-based diagnosis of virus infection could significantly reduce the unnecessary exposure to contagious persons and support real-time monitoring and tracing of infectious pathogens. In addition, it may alleviate the pressure of excessive confirmatory tests and reduce the burden on the hospitals, while allowing individuals a diagnosis method that can be used at home, point of care (PoC), and central facilities.

Benefiting from the assistance of machine learning analysis, our pathogenic infection diagnosis system (PIDS) showed prediction capability from breath that may help decision-makers and healthcare systems improve the way the COVID-19 information is approached. It could reduce time, cost, and number of unneeded confirmatory tests, lowering the burden on hospitals. During the hospitalization and at-home isolation, the PIDS may act as a monitoring tool for evaluating the treatment efficacy and disease regression. When apply the PIDS to a large number of users, it is feasible to create a sample database and establish a learning model for predicting the disease development among the high-risk groups, and hospitalization periods and prognosis for positive patients. It may enable not only the individual diagnosis, treatment, and follow-up, but also continual screening of at-risk populations and real-time monitoring of epidemics[20].

Supplementary Table 3. Information of 21 negative cases. Source data are provided as a Source Data file.

No.	Age	Gender	RR	EBT	Infection	Vaccination
1	26	Female	17	32.3	Yes (recovered)	BNT×3
2	24	Female	18	32.8	No	BNT×3
3	23	Female	18	32.8	No	BNT ×3
4	27	Male	20	32.5	Yes (recovered)	Sinovac×3
5	21	Female	16	32.7	No	BNT ×3
6	22	Male	17	32.5	No	Sinovac×2， BNT×1
7	19	Male	17	32.7	Yes (recovered)	BNT ×3
8	20	Female	17	32.9	No	Sinovac×2, BNT×1
9	19	Female	15	32.4	No	BNT ×3
10	22	Female	16	32.5	No	BNT ×3
11	21	Female	18	33.0	Yes (recovered)	BNT ×3
12	21	Female	19	32.3	No	BNT ×3
13	26	Female	14	32.7	No	BNT ×3
14	20	Male	18	32.2	No	BNT ×3
15	19	Male	15	33	No	BNT ×3
16	30	Female	19	32.8	No	Sinovac×3
17	31	Male	18	32.3	No	Sinovac×3
18	26	Male	18	32.5	Yes (recovered)	BNT×3
19	28	Male	16	32.7	No	Sinovac×2
20	27	Male	16	32.6	No	Sinovac×3
21	30	Male	17	32.9	No	Sinovac×3

Notes: Comirnaty is also called mRNA vaccine (BNT). Sinovac is also called Vero Cell vaccine.

Supplementary Table 4. Information of 21 positive cases. Source data are provided as a Source Data file.

No.	Age	Gender	RR	EBT	Symptom	Vaccination	Medicine
1	34	Male	21	33.3	sore throat; sore muscle; no fever; (mild)	BNT×3	Lianhua Qingwen capsule
2	35	Male	23	33.2	sore throat; no fever; (mild)	BNT×3	Panadol
3	34	Female	17	32.9	sputum; no fever; (near asymptomatic)	BNT×3	Panadol
4	30	Male	22	33.7	sore throat; no fever; (mild)	BNT×2	Prescription medicine (expectorant, antipyretic)
5	34	Female	27	34.7	terrible sore throat; bad cough; pant; taste loss; heavy headache; nasal obstruction; high fever; (severe)	BNT×3	Prescription medicine (expectorant, antipyretic)
6	31	Female	25	34	high fever, cold, sore muscle, terrible sore throat, bad cough, heavy headache；dizzy; (severe)	BNT×3	Without medicine; Three supplements: grapefruit seed extract, ester-C, Zinc Chelate
7	55	Female	16	32.3	sputum；no fever; (near asymptomatic)	BNT×3	Panadol, Vitamin C
8	36	Male	19	32.7	runny nose; no fever; (near asymptomatic)	BNT×3	CLOFENAC SR (Diclofenac SR tablet 100 mg)
9	28	Male	26	34.5	terrible sore throat; bad cough; runny nose; dizzy; nausea; high fever; (severe)	BNT×3	Lianhua Qingwen capsule
10	42	Male	22	33.8	runny nose; cold sweat; weak; no fever; (mild)	None	Panadol
11	31	Female	23	33.5	sore throat; cough; headache; weak; no fever; (mild)	None	Without medicine
12	24	Male	25	34.2	bad cough; runny nose; terrible Sore throat, dizzy, high fever; (severe)	Sinovac×2	Panadol, Cough syrup, Ryukakusan
13	21	Female	26	34.6	high Fever, terrible Sore throat; Pant; bad Cough; Runny nose; dizzy, heavy Headache; Joint pain；taste loss; (severe)	Sinovac×2	Panadol, Cough syrup, Ryukakusan
14	57	Male	24	34	high Fever; Sore throat; bad cough; runny nose; taste loss; (severe)	Sinovac×2	Panadol, Cough syrup, Ryukakusan
15	27	Female	26	34.5	terrible sore throat; runny nose; pant, bad cough; sputum; dizzy high fever; (severe)	BNT×3	Prescription medicine (Anti-inflammatory drug; Cold medicine)
16	23	Female	23	33.2	sore throat; cough; runny nose; no fever; (mild)	BNT×3	Panadol
17	26	Female	21	33.7	sore throat; cough; runny nose; no fever; (mild)	BNT×3	Panadol
18	26	Male	25	34.2	high fever; bad cough; runny nose; pant; terrible sore throat; (severe)	BNT×3	Panadol, Expectorant, COLTALIN
19	53	Male	18	32.6	runny nose; no fever; (near asymptomatic)	BNT×3	Cough syrup, Cold medicine
20	53	Female	17	32.3	runny nose; no fever; (near asymptomatic)	Sinovac×3	Cough syrup, Cold medicine
21	28	Female	18	32.5	itchy throat; no fever; (near asymptomatic)	Sinovac×2, BNT×1	Lianhua Qingwen capsule

Supplementary Figures

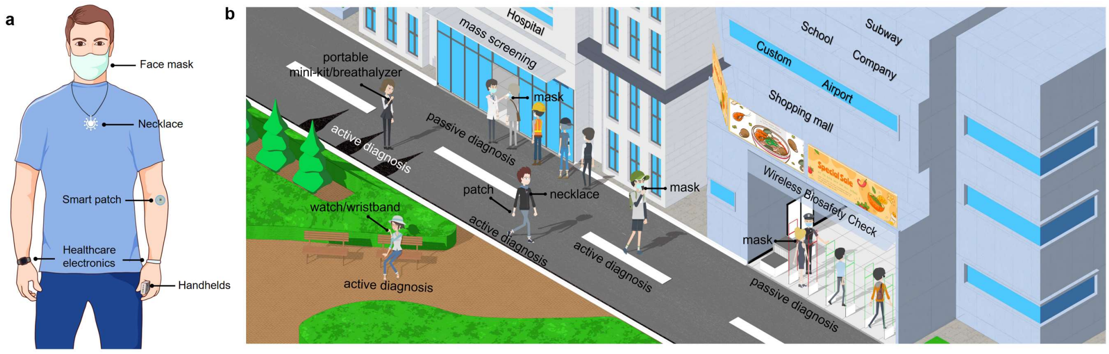

Supplementary Fig. 1| Outlook of diagnosis for virus infection by breath and blow using the wireless, battery-free, miniaturized, evolvable, multifunctional PIDS. a Compared with the conventional detections by saliva and swabbed samples in clinical settings, breath and blow provided preferred access to respiratory pathogens. The miniaturized wearable and handhelds made pandemic diagnosis smarter and more convenient. For example, the combination with face mask, adornment (necklace), smart patch, watch, wristband, and handheld rapid detection kit. These products fully guaranteed the convenience and choosability in multiple life scenarios. **b** Wireless, battery-free, active self-diagnosis and passive screening and diagnosis in multiple life scenarios, including at-home test, outdoors, hospitals and public gathering places installed with biosafety doors. Individuals can use the evolved PIDS to conduct active self-diagnosis anywhere at any time. Medical workers can rapidly conduct passive mass screening using the smartphones, greatly saving detection time and reducing the risk of cross infection. The public gathering place (e.g., subway, school, company, custom, airport, shopping mall) can rapidly screen and diagnose the infected individuals by the wireless biosafety doors.

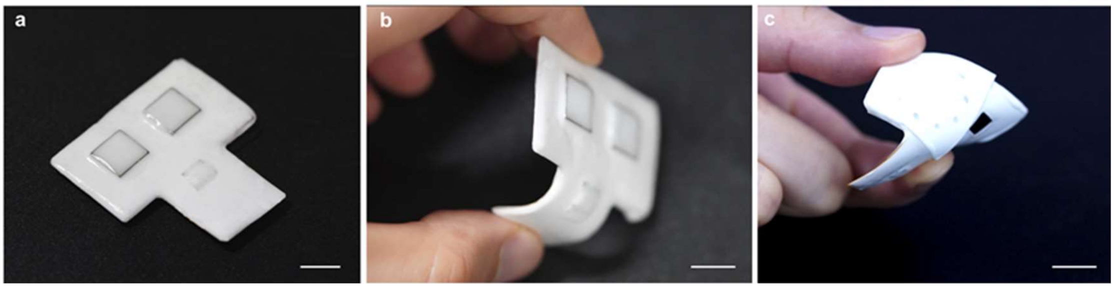

Supplementary Fig. 2| Pictures of the packaged PIDS in different states. a Back view without bending. **b** Back view with bending. **c** Front view with bending. Scale bar: 1 cm.

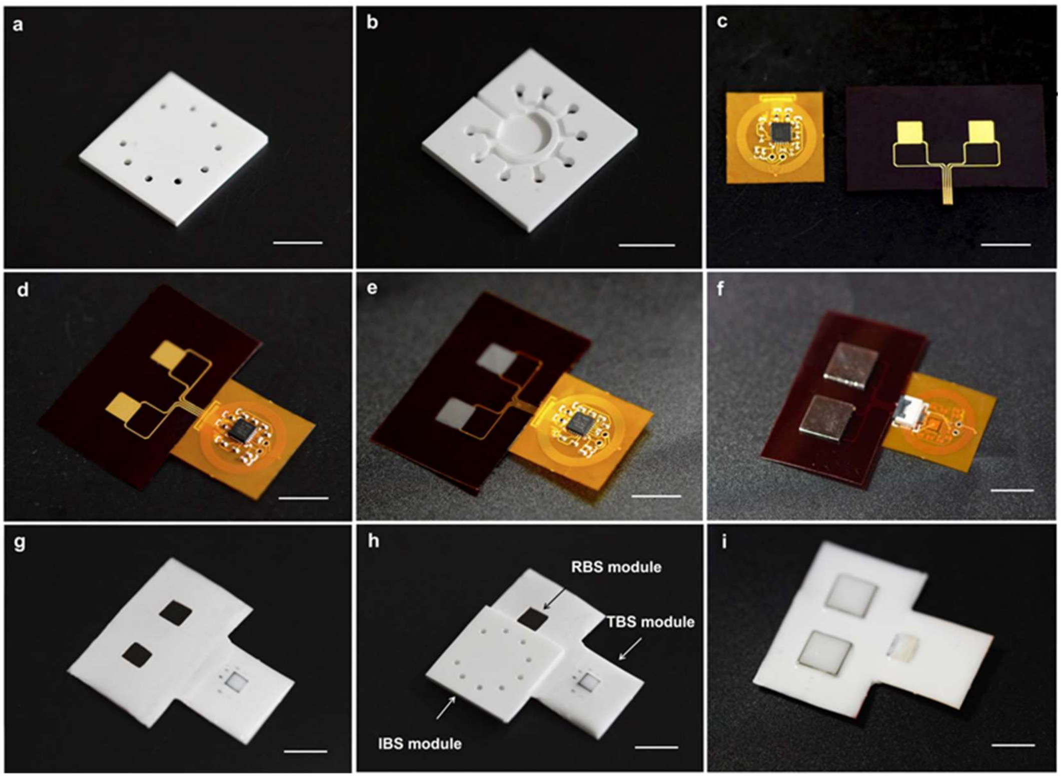

Supplementary Fig. 3| Preparation process of the multifunctional PIDS for monitoring the exhaled virus, respiration rate and exhaled breath temperature. a Front view of the coronavirus-inspired microchannel. **b** Back view of the coronavirus-inspired microchannel. **c** Pictures of the back-end NFC circuit and front-end interdigital electrodes on PI film. **d** Picture of connecting the back-end NFC circuit with the front-end interdigital electrodes. **e** Graphene was sprayed onto the interdigital electrodes. **f** Back view of the PI film and NFC circuit. Two steel sheets (1cm × 1cm × 1mm) were fixed on the backs of IBS module and RBS module to protect them from external mechanical interference. **g** The front-end biosensing modules were connected to the back-end NFC circuits and packaged with white PDMS. **h** A bionic microchannel was covered on the IBS module. The acted as the IBS module. The right bare graphene area acted as the RBS module. The TBS module was built in the back-end NFC circuit. **i** Back view of the packaged PIDS with white PDMS. Scale bar: 1 cm.

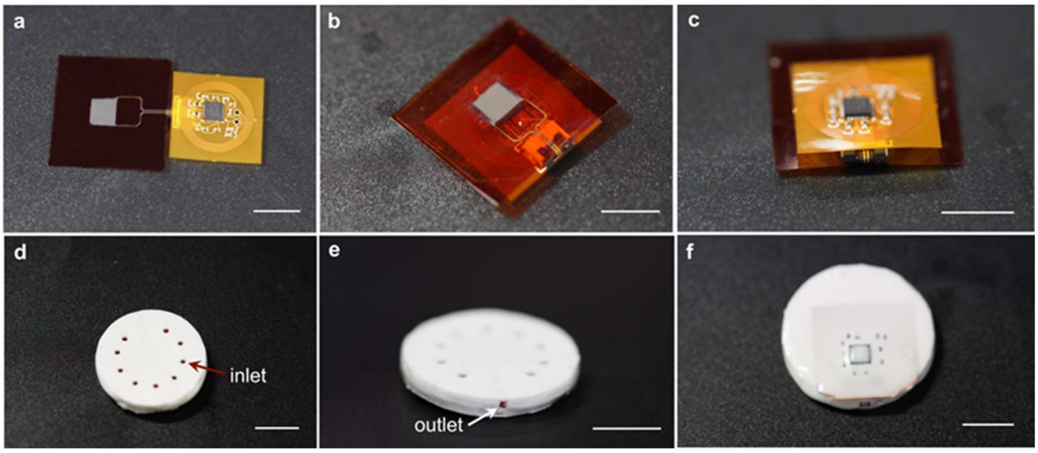

Supplementary Fig. 4| Preparation process of the circular PIDS. The RBS module was removed to guarantee the evolution ability of PIDS. The derived PIDS focused on the virus detection. These products collected virus sample by blow. **a** Picture of a flat PIDS without package. This PIDS can be directly packaged using PDMS with white pigment and covered with the bionic microchannel. Such as the handheld breathalyzer. **b, c** The NFC circuit is placed on the back of the PI film. **b** Front view. **c** Back view. This PIDS can be directly packaged using PDMS with white pigment and covered with the square microchannel. Such as the mini-kit, watch and wristband. **d-f** Similarly, if the PIDS in **b** and **c** is covered with a circular multilevel channel, it can be derived into a smart patch or a necklace. **d,e** Front views of the sample with inlets and outlets, respectively. **f** Back view of the PIDS. The edge was properly trimmed to keep the circular shape. Scale bar: 1 cm.

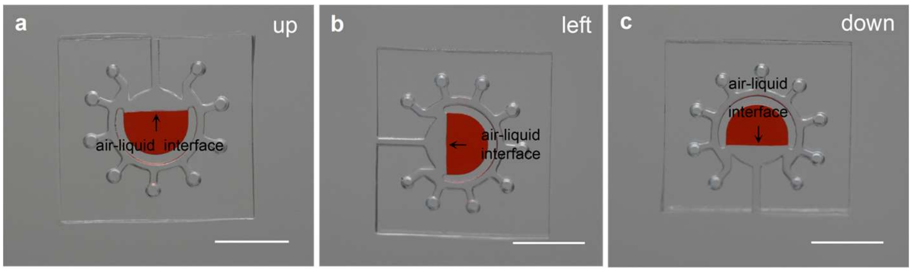

Supplementary Fig. 5| The hydrophobicity of PDMS contributed to keeping the air-liquid interface stable in different directions. a Up. **b** Left. **c** Down. The PBS solution in the chamber keeps stable without flowing out at any directions. Scale bar: 1 cm.

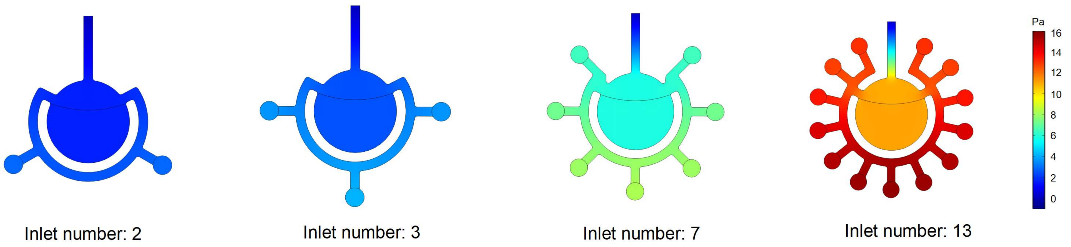

Supplementary Fig. 6| Simulation of the air-liquid interface pressure with different inlet numbers in the symmetrical stable condition. The airflow velocity of inlets was set as 0.01 m/s. The pressure increased with the inlet numbers from 2 to 13. A small number of inlets allowed small amounts of air to flow into the chamber and produced a lower pressure at the air-liquid interface. A large number of inlets allowed large amounts of air to flow into the chamber and produced a higher pressure at the air-liquid interface.

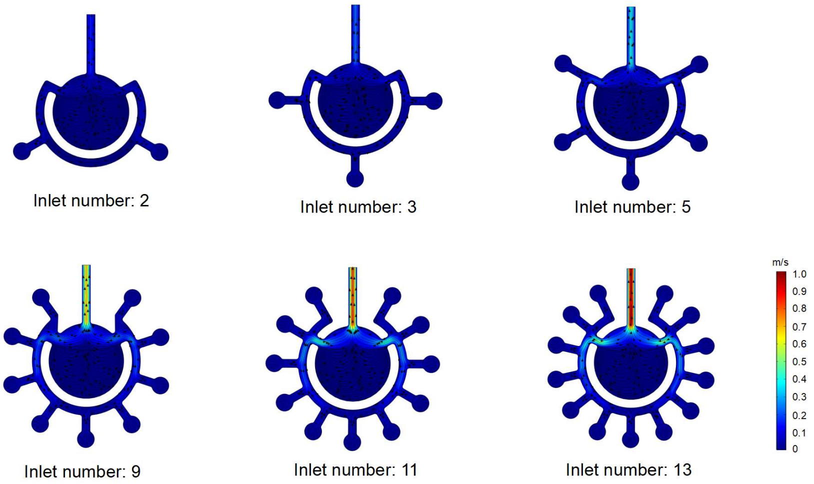

Supplementary Fig. 7| Simulation of airflow velocity at the outlet end with different inlet numbers in the symmetrical stable condition. The airflow velocity of inlets was set as 0.01 m/s. The airflow velocity of outlet increased with the inlet number. A small number of inlets allowed small amounts of air to flow into the chamber and produced a slower airflow at the outlet. A large number of inlets allowed large amounts of air to flow into the chamber and produced a faster airflow at the outlet.

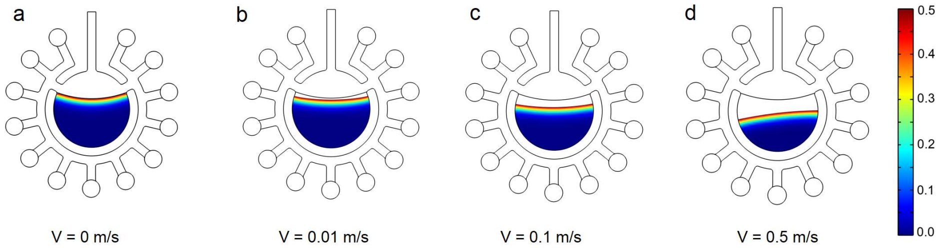

Supplementary Fig. 8| Simulation of volume fraction of PBS with different airflow velocities in the asymmetrical transient condition. With the velocity increased gradually, some of the liquids were beyond the threshold value (0.5), the air-liquid interface lowered gradually. This part of liquid can be regarded as disappeared. **a** Primary state of the liquid without blow. All the airflow velocities are 0 m/s. **b-d** The airflow velocities of the upper left two inlets are 0.01 m/s, 0.1 m/s and 0.5 m/s, respectively. The airflow velocities of other inlets are all 0.01 m/s. The results are captured at 0.3 s.

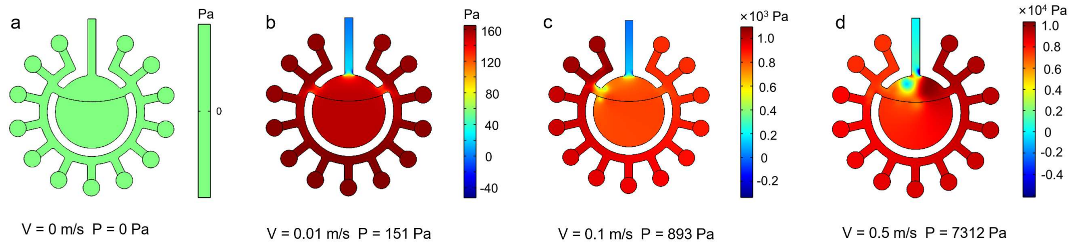

Supplementary Fig. 9| Simulation of the air-liquid interface pressure with different airflow velocities in the asymmetrical transient condition. With the velocity increased gradually, the pressure in the chamber also increased gradually. **a** Primary state of the liquid without blow. All the airflow velocities are 0 m/s. **b-d** The airflow velocities of the upper left two inlets are 0.01 m/s, 0.1 m/s and 0.5 m/s, respectively. The airflow velocities of other inlets are all 0.01 m/s. The results are captured at 0.3 s.

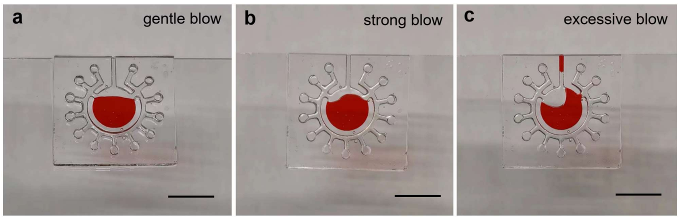

Supplementary Fig. 10| Liquid change in chamber with different strength of blow. The microchannel has 13 inlets and 1 outlet around the chamber. **a** The air-liquid interface kept stable with the gentle blow. **b** The air-liquid interface showed a fluctuated interface with a strong blow. **c** The liquid was blown away with an excessive blow. Scale bar: 1 cm.

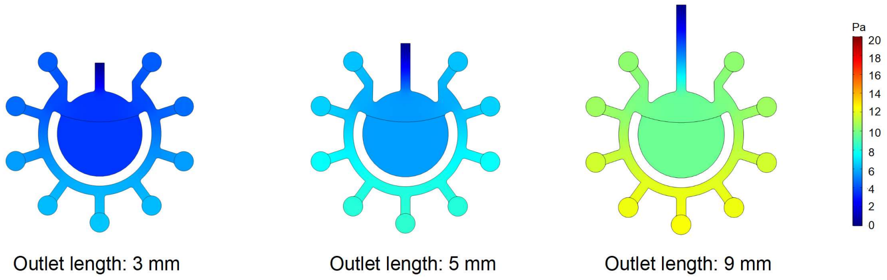

Supplementary Fig. 11| Simulation of the air-liquid interface pressure with different outlet lengths in the symmetrical stable condition. The airflow velocity of inlets was set as 0.01 m/s. The pressure in chamber increased with the outlet lengths. A short length allows the faster airflow to emit out from chamber and produce a lower pressure in the chamber. A long length allows the slower airflow to emit out from the chamber and produces a higher pressure in the chamber.

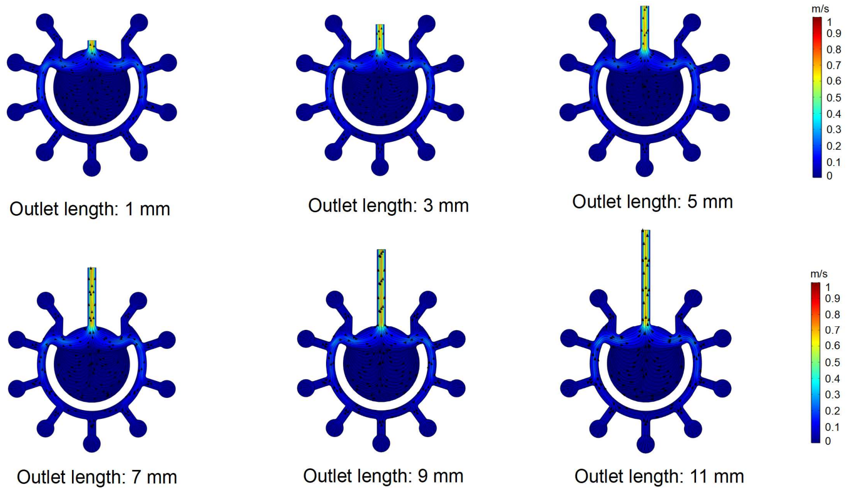

Supplementary Fig. 12| Simulation of airflow velocity at the outlet end with different outlet lengths in the symmetrical stable condition. The airflow velocity of inlets was set as 0.01 m/s. The airflow velocity decreased with the outlet lengths. A short length allows the faster airflow to emit out from chamber due to its smaller volume. A long length allows the slower airflow to emit out from the chamber due to its larger volume.

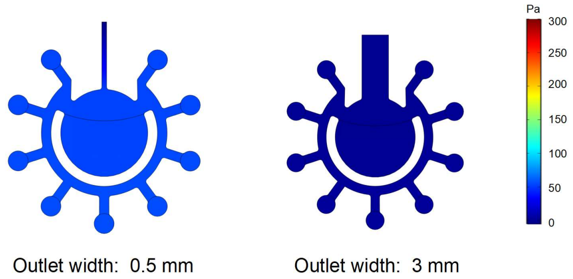

Supplementary Fig. 13| Simulation of the air-liquid interface pressure with different outlet widths in the symmetrical stable condition. The airflow velocity of inlets was set as 0.01 m/s. The pressure decreased with the outlet widths. When the outlet height (1mm), outlet number (9) and outlet length (7 mm) were fixed, the narrow and wide widths meant the smaller and larger outlet volumes, respectively. The narrow outlet caused a smaller emission volume at the same airflow velocity, which in turn retained more air in the chamber within the same time and further increased the chamber pressure. The wide outlet induced a larger emission volume at the same airflow velocity, which in turn retained less air in the chamber and further decreased the pressure in chamber.

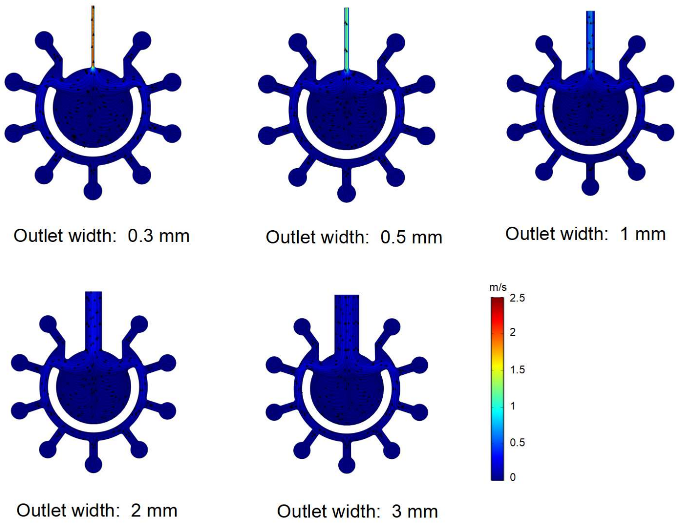

Supplementary Fig. 14| Simulation of airflow velocity at the outlet end with different outlet widths in the symmetrical stable condition. The airflow velocity of inlets was set as 0.01 m/s. The airflow velocity at the outlet end decreases with the outlet width. When the outlet height (1mm), outlet number (9) and outlet length (7 mm) were fixed, if the same volume of air flowed into the outlet within the same time, it will cause a faster airflow velocity in the narrow outlet and slower airflow velocity in the wide outlet.

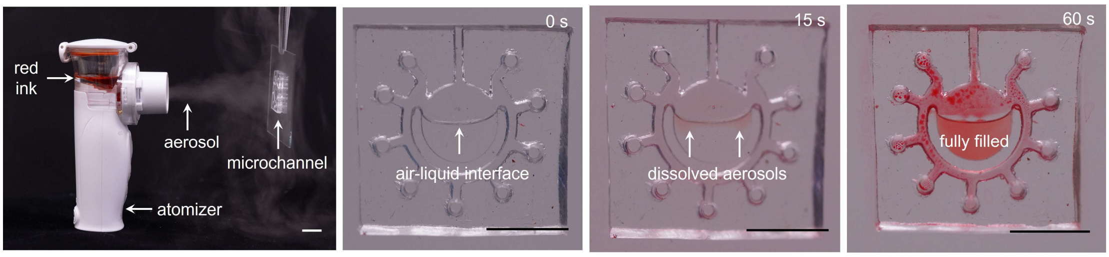

Supplementary Fig. 15| Simulation of the rapid collection of breath sample and blow with an atomizer. The microchannel collected the aerosols within 15 s through the air-liquid interface. The distance between the atomizer and microchannel was zero. The red aerosols fully filled the PBS solution within 60 s showing its outstanding ability of collecting aerosol sample in breath and blow. Scale bar: 1 cm.

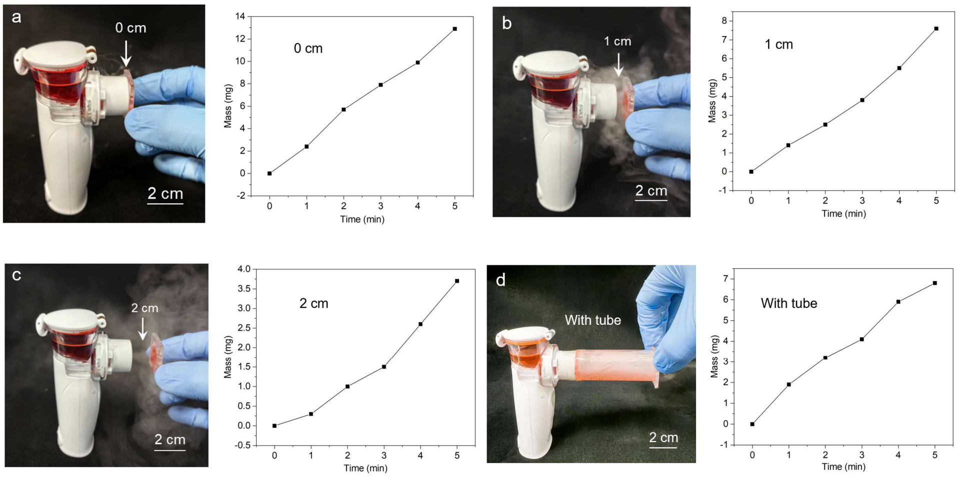

Supplementary Fig. 16| Collection simulation of aerosols using an atomizer. a-c The microchannel was placed at different positions. The distances between the atomizer and microchannel were (**a**) 0 cm, (**b**) 1 cm, and (**c**) 2 cm, respectively. The collection speed decreased with the increased distance. **d** A tube was added as a controlling condition to test the collection speed of aerosols. The distance between the tube and microchannel was 0 cm. Source data are provided as a Source Data file.

We added a tube as the controlling environment to explore its influence on the collection and viral capturing speed at the air-liquid interface. The height, diameter and thickness of the tube were 6 cm, 2.2 cm, and 1 mm, respectively. The environment temperature and ink temperature are all 25 °C. As shown in supplementary Fig. 16, the collection speed decreased with the distance from 0 cm to 2 cm (Fig. 16a to 16c), which can be attributed to the decreased airflow velocity with the distance. When a tube was added as the controlling condition (supplementary Fig. 16d), the collection speed was slower than that without the tube at 0 cm distance in supplementary Fig. 16a, but faster than that without tube at 2 cm distance in supplementary Fig. 16c. The results indicated that the enclosed space provided by the spray nozzle of the atomizer (supplementary Fig. 16a) and the tube (supplementary Fig. 16d) help to collect the aerosols, which could be attributed to the higher pressure in the enclosed space. Meanwhile, the longer the distance, the slower the collection speed.

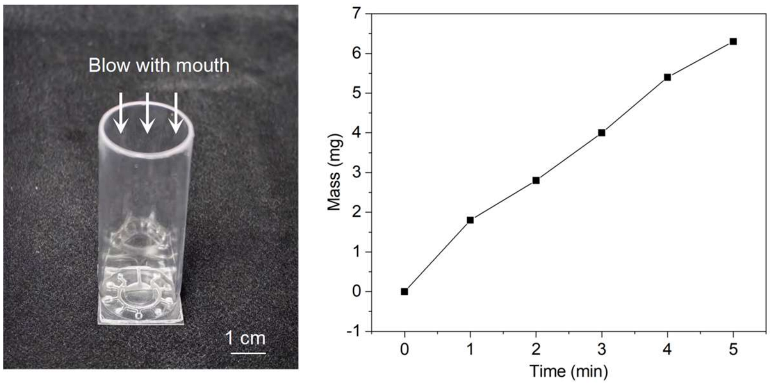

Supplementary Fig. 17| Collection speed of the aerosols with mouth blow. The tube was added on the microchannel and covered the inlets. The mouth directly blew the upper end of the tube. The distance between mouth and the upper end of the tube was 1 cm. The blow frequency was 20 times per minute. The time was about 3 seconds for each blow cycle. Source data are provided as a Source Data file.

Compared with the mouth blow without the tube in the manuscript (human blow, Fig. 2o), the collection speed with the tube by mouth blow became slower. The results can be attributed to the temperature difference between human blow and the environment. Considering that the airflow velocity by mouth blow is much faster than that of atomizer, the velocity attenuation in the tube (6 cm in length) can be neglected. However, the temperature of mouth blow is higher (about 32.5 °C) than the environment temperature (25 °C), the temperature difference in the open environment without tube help to condense the exhaled aerosols and accelerate its collection. On the contrary, the tube provided an enclosed space that confined the airflow in the tube and reduced the temperature difference. It is unfavorable for condensing and collecting the aerosols. This could be the main reason for the slower collection speed in an enclosed space.

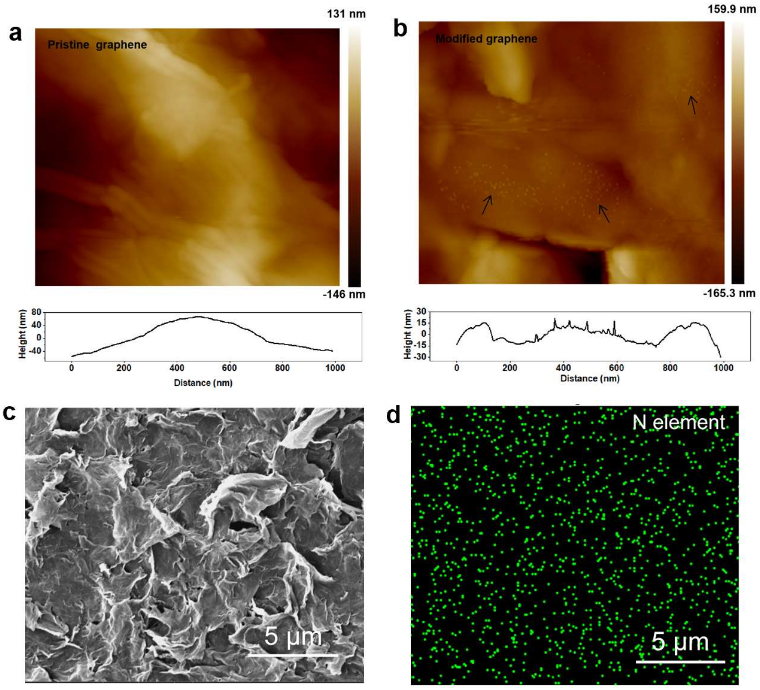

Supplementary Fig. 18| Surface morphologies and characteristic elements of the activated graphene. a, b Surface morphologies before and after modification with PBASE, respectively. They are characterized using the atomic force microscope (AFM). Some nanoparticles formed on graphene surface after modification (black arrows). **c, d** Surface morphologies and characteristic element (N, nitrogen) of the activated graphene.

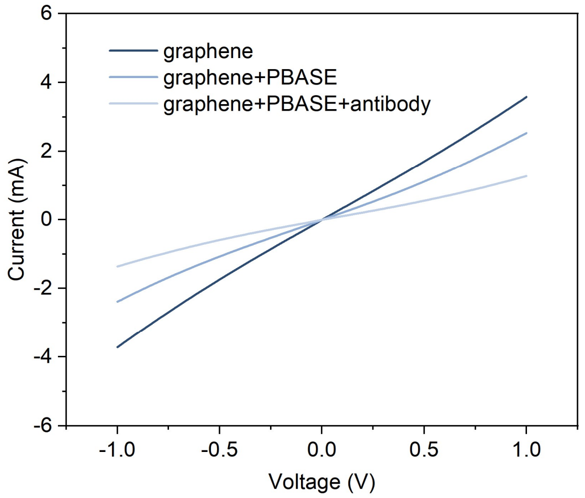

Supplementary Fig. 19| Current change after modifying PBASE and the spike antibody. The scan range is from -1 V to +1 V using the semiconductor analyzer. The current gradually decreases after modifying the molecular linker and antibody, which proves the efficient modification of PBASE and the antibody on graphene. Source data are provided as a Source Data file.

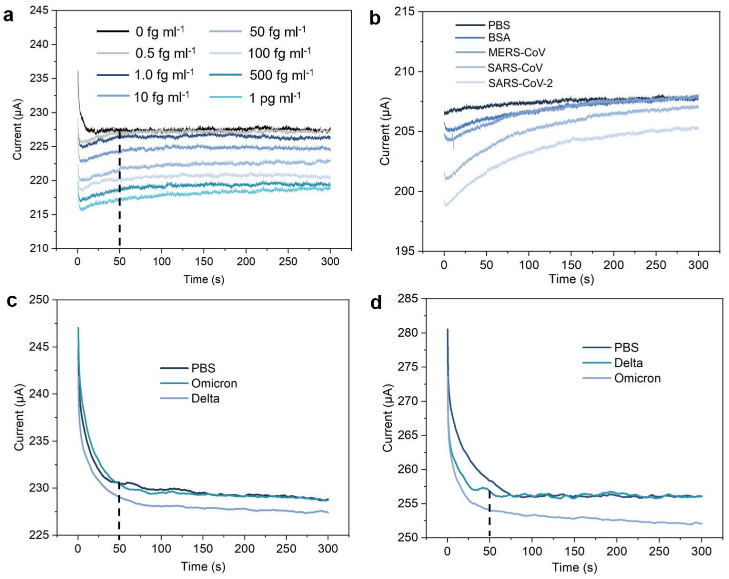

Supplementary Fig. 20| Limit of detection (LOD), specific identification and selective identification of IBS module to SARS-CoV-2 spike proteins by using different antibodies. a LOD of the IBS module modified with antibody 1 (spike S1 antibody, Cat: 40150-R007) to SARS-CoV-2 spike protein. The concentration range was between 1 fg ml^{-1} and 1 pg ml^{-1}. **b** Specific identification of antibody 1 to SARS-CoV-2 spike protein. The antigen proteins included BSA, MERS-CoV, SARS-CoV and SARS-CoV-2. **c, d** Selective identification of the pandemic SARS-CoV-2 variants, i.e., Delta and Omicron. Antibody 2 (Cat:40592-MM57) binds to Delta but not to Omicron. It showed a rapid response to Delta variant within 50 s and no response to Omicron variant. Antibody 3 (Cat:40591-MM48) can selectively recognize Omicron. It showed a rapid response to Omicron variant within 50 s and no response to Delta. The test concentration was 500 fg ml^{-1}. These results indicated that the IBS module can not only detect SARS-CoV-2 variants, but also identify the virus species by modifying different antibodies. Source data are provided as a Source Data file.

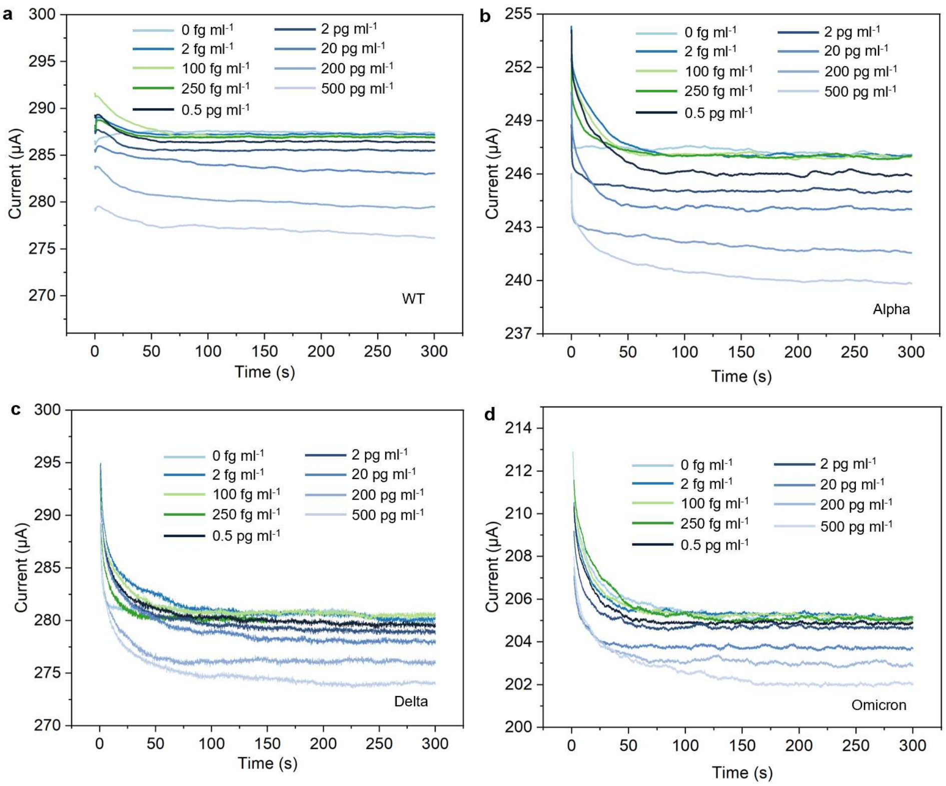

Supplementary Fig. 21| Detection performance of the IBS module to SARS-CoV-2 pseudo virus including WT, Alpha, Delta and Omicron. Antibody 1 was modified on graphene surface. The test concentration range is between 2 fg ml^{-1} and 500 pg ml^{-1}. **a** WT. **b** Alpha. **c** Delta. **d** Omicron. IBS showed no response to the pseudo viruses until the concentration reached to 0.5 pg ml^{-1}. All the currents decreased gradually with the concentration, indicating that the IBS module was capable of rapidly detecting SARS-CoV-2 variants. Additionally, when the concentration of pseudo viruses was lower than the LOD (0.5 pg ml^{-1}), the response current curves overlapped with the baseline current, indicating that IBS was stable without drift during the test. When the concentration was very high (e.g., 500 pg ml^{-1}), the response current decrease gradually with time, which was because that the efficient dynamic binding between the antibody and spike protein of pseudo viruses was enhanced gradually with incubation time. More pseudo viruses can bind with the antibody over time. Source data are provided as a Source Data file.

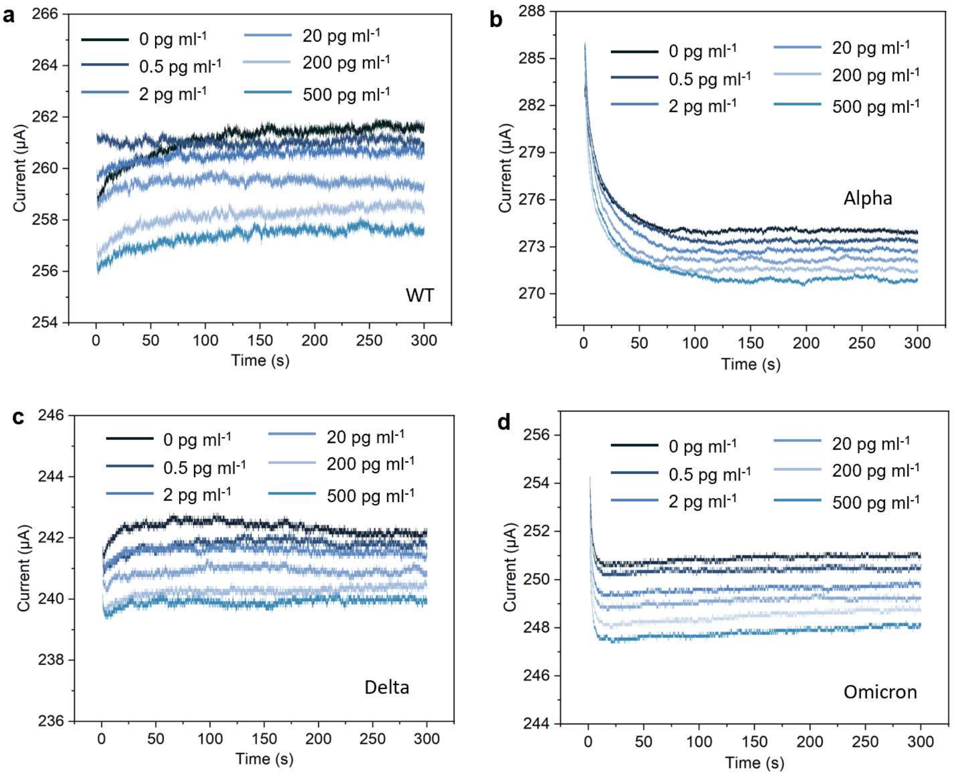

Supplementary Fig. 22| Detection performance of the IBS module to the live viruses including WT, Alpha, Delta and Omicron. Antibody 1 was modified on graphene surface. The concentration range is between 2 fg ml^{-1} and 500 pg ml^{-1}. **a** WT. **b** Alpha. **c** Delta. **d** Omicron. Source data are provided as a Source Data file.

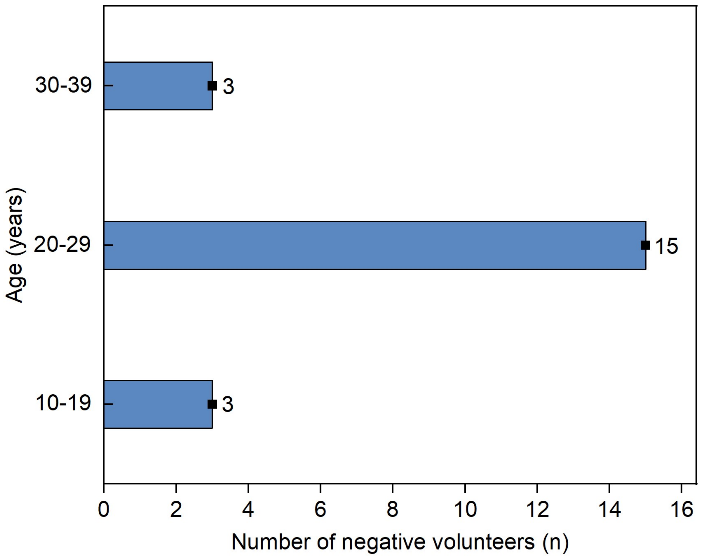

Supplementary Fig. 23| Ages of the 21 negative participants. Age distribution of the negative participants. Three cases are between 30 and 39. Fifteen cases are between 20 and 29. Three cases are between 10 and 19. Source data are provided as a Source Data file.

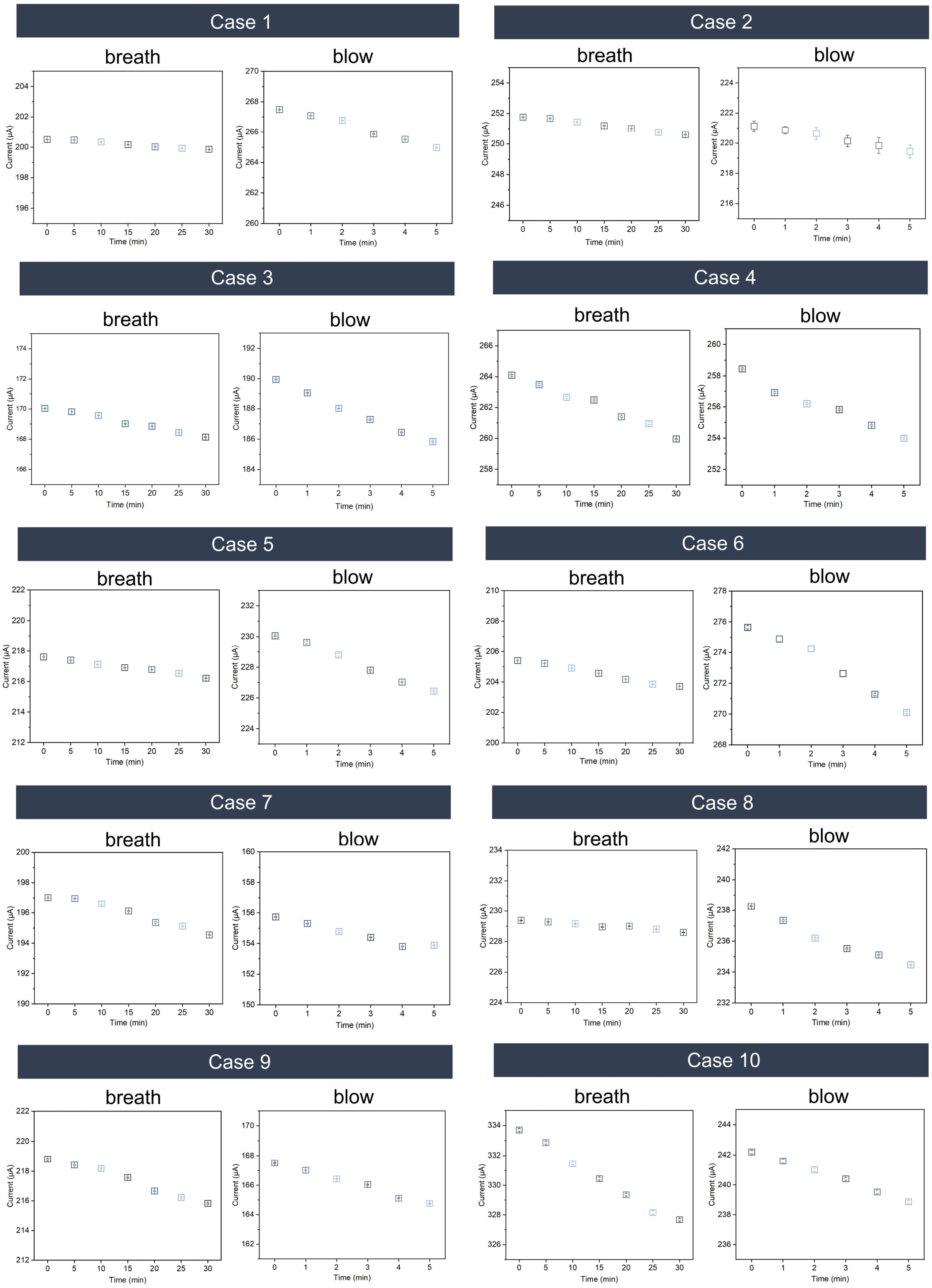

Supplementary Fig. 24| Data of the positive case 1 to case 10. Case 3 was used in Fig. 4e and Fig. 4f in the manuscript. The error bars correspond to the standard deviation (SD). The average current values and error bars were calculated using the recorded 1200 stable current values (n=1200) of each test. Data are presented as mean values ± SD. Source data are provided as a Source Data file.

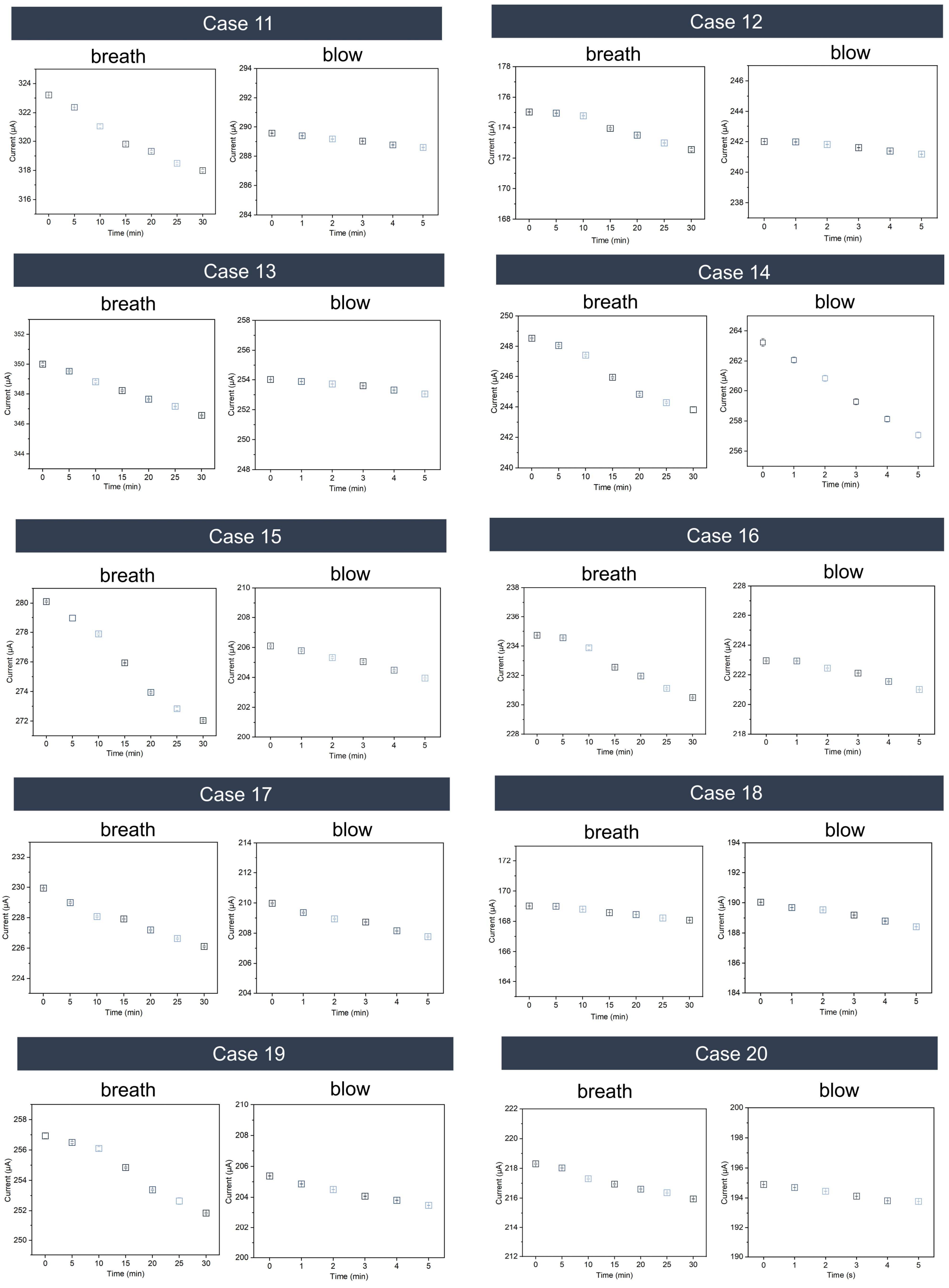

Supplementary Fig. 25| Data of the positive case 11 to case 20. The error bars correspond to the standard deviation. The average current values and error bars were calculated using the recorded 1200 stable current values (n=1200) of each test. Data are presented as mean values ± SD. Source data are provided as a Source Data file.

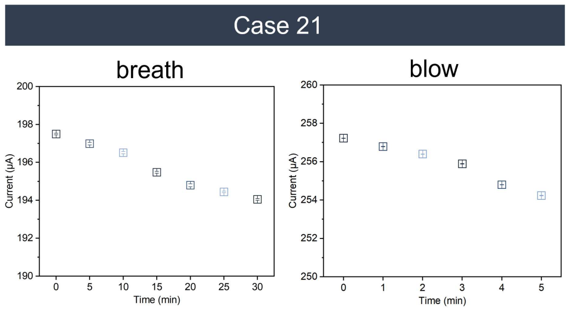

Supplementary Fig. 26| Data of the positive case 21. The error bars correspond to the standard deviation. The average current values and error bars were calculated using the recorded 1200 stable current values (n=1200) of each test. Data are presented as mean values ± SD. Source data are provided as a Source Data file.

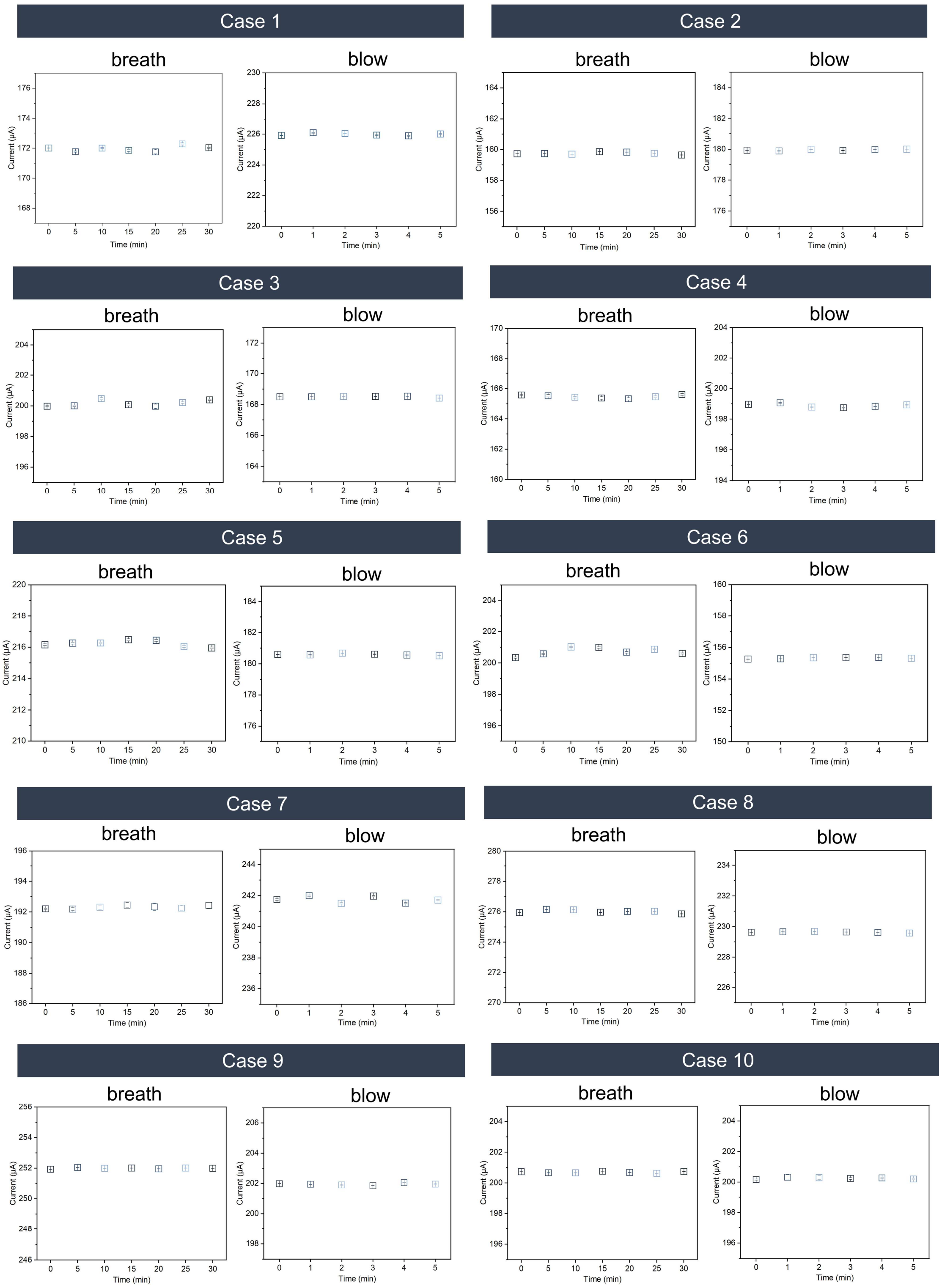

Supplementary Fig. 27| Data of the negative case 1 to case 10. Case 1 was used in Fig. 4g and Fig. 4h in the manuscript. The error bars correspond to the standard deviation. The average current values and error bars were calculated using the recorded 1200 stable current values (n=1200) of each test. Data are presented as mean values ± SD. Source data are provided as a Source Data file.

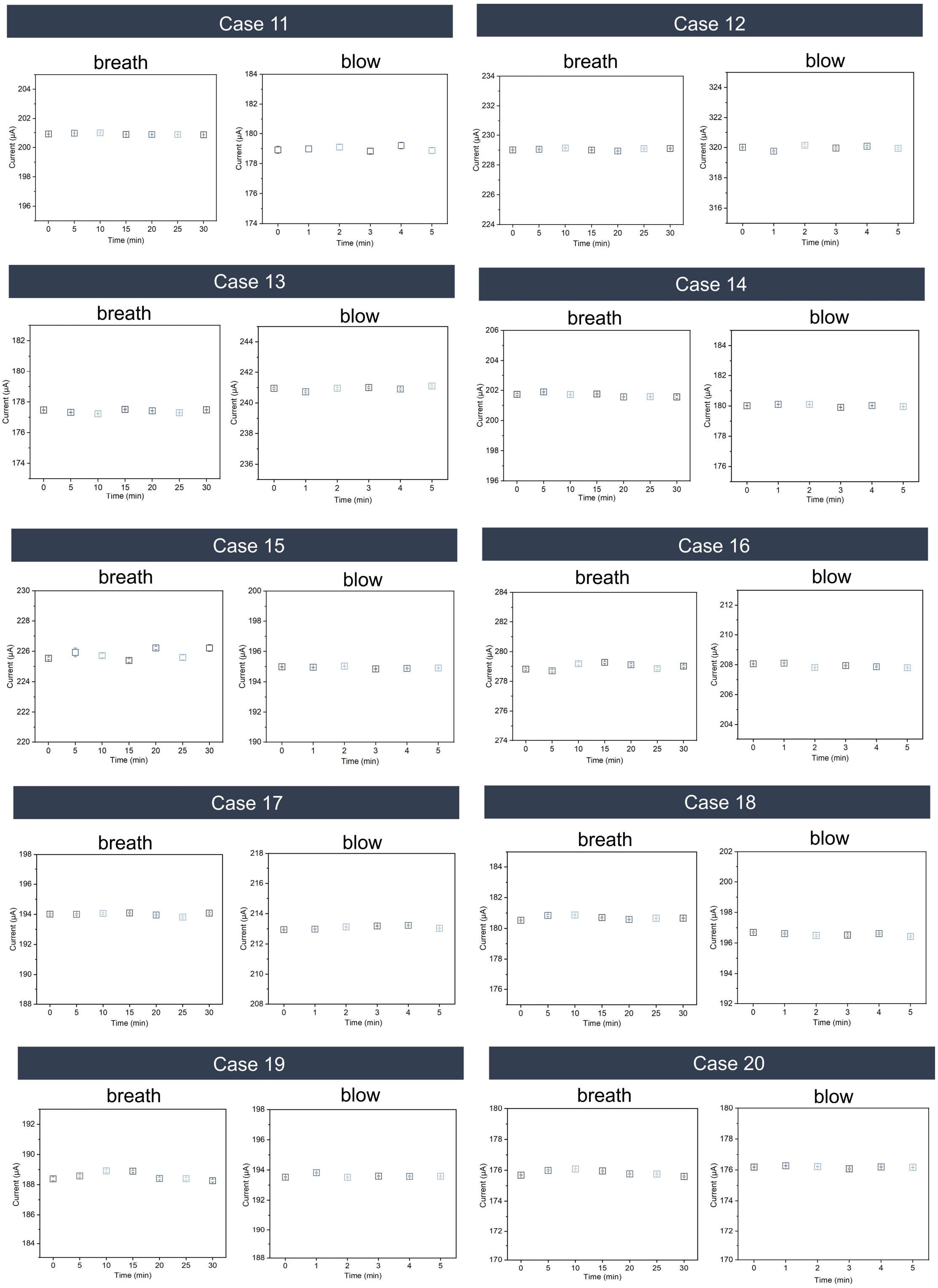

Supplementary Fig. 28| Data of the negative case 11 to case 20. The error bars correspond to the standard deviation. The average current values and error bars were calculated using the recorded 1200 stable current values (n=1200) of each test. Data are presented as mean values ± SD. Source data are provided as a Source Data file.

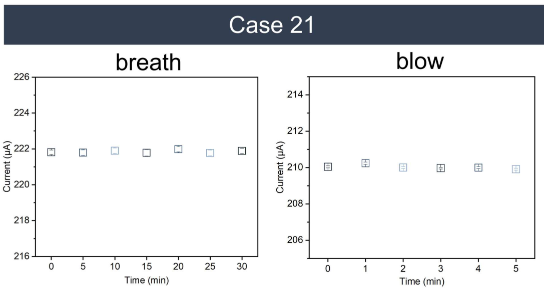

Supplementary Fig. 29| Data of the negative case 21. The error bars correspond to the standard deviation. The average current values and error bars were calculated using the recorded 1200 stable current values (n=1200) of each test. Data are presented as mean values ± SD. Source data are provided as a Source Data file.

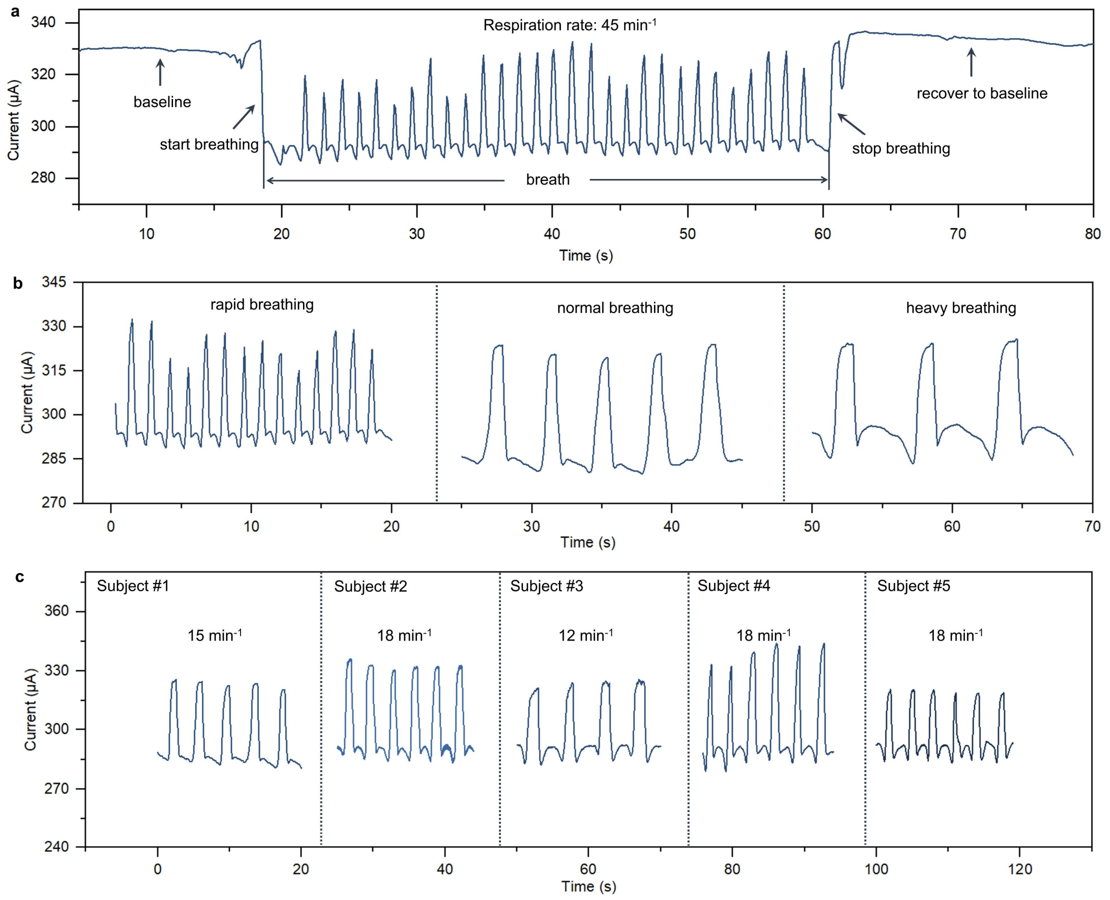

Supplementary Fig. 30| Respiratory characteristics recorded by the RBS module. **a** A typical breath curve recorded by the RBS module with a rapid respiration rate (45 min^{-1}). The RBS module showed a sensitive response to the adsorption and desorption of aerosols, which caused periodic decrease and increase of the current. **b** Respiration curves of the rapid breathing, normal breathing, and heavy breathing. **c** General applicability of the RBS module to different individuals, proving its practicability in recording respiratory characteristics. Source data are provided as a Source Data file.

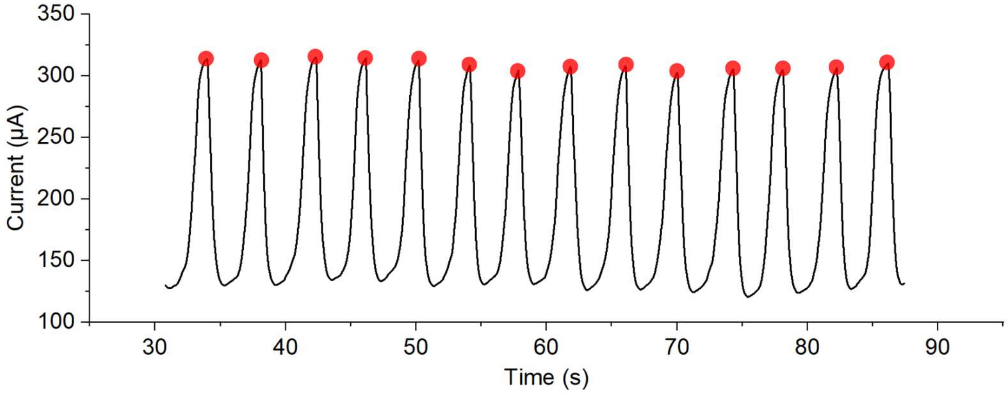

Supplementary Fig. 31| Respiratory rate (RR) calculation of a regular breath curve. There are 14 peaks from 34 s to 86.2 s. The RR can be calculated as $RR = \frac{60\ s}{(86.2-34)s} \times 14 \approx 16\ min^{-1}$. This data was from the negative case 4 in supplementary Fig. 33. Source data are provided as a Source Data file.

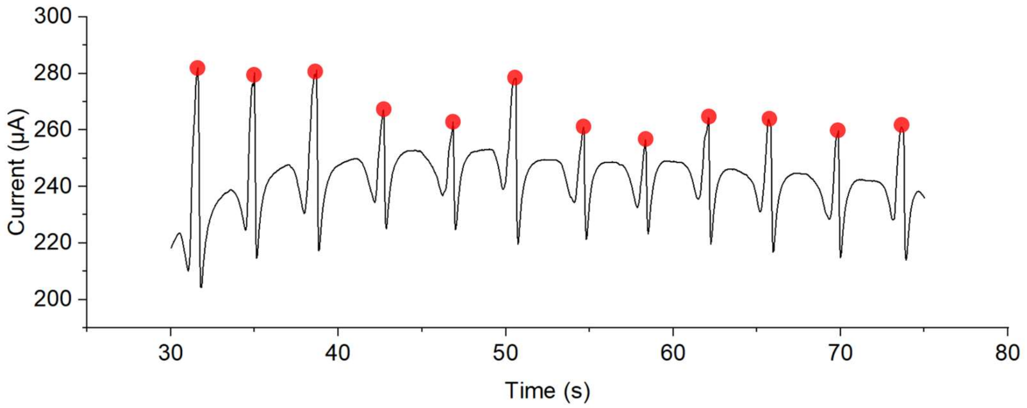

Supplementary Fig. 32| Respiratory rate (RR) calculation of an irregular breath curve. There are 12 peaks from 31.6 s to 79.78 s. The RR can be calculated as $RR = \frac{60s}{(73.6-31.6)s} \times 12 \approx 17\ min^{-1}$. This data was from the positive case 2 in supplementary Fig. 37. Source data are provided as a Source Data file.

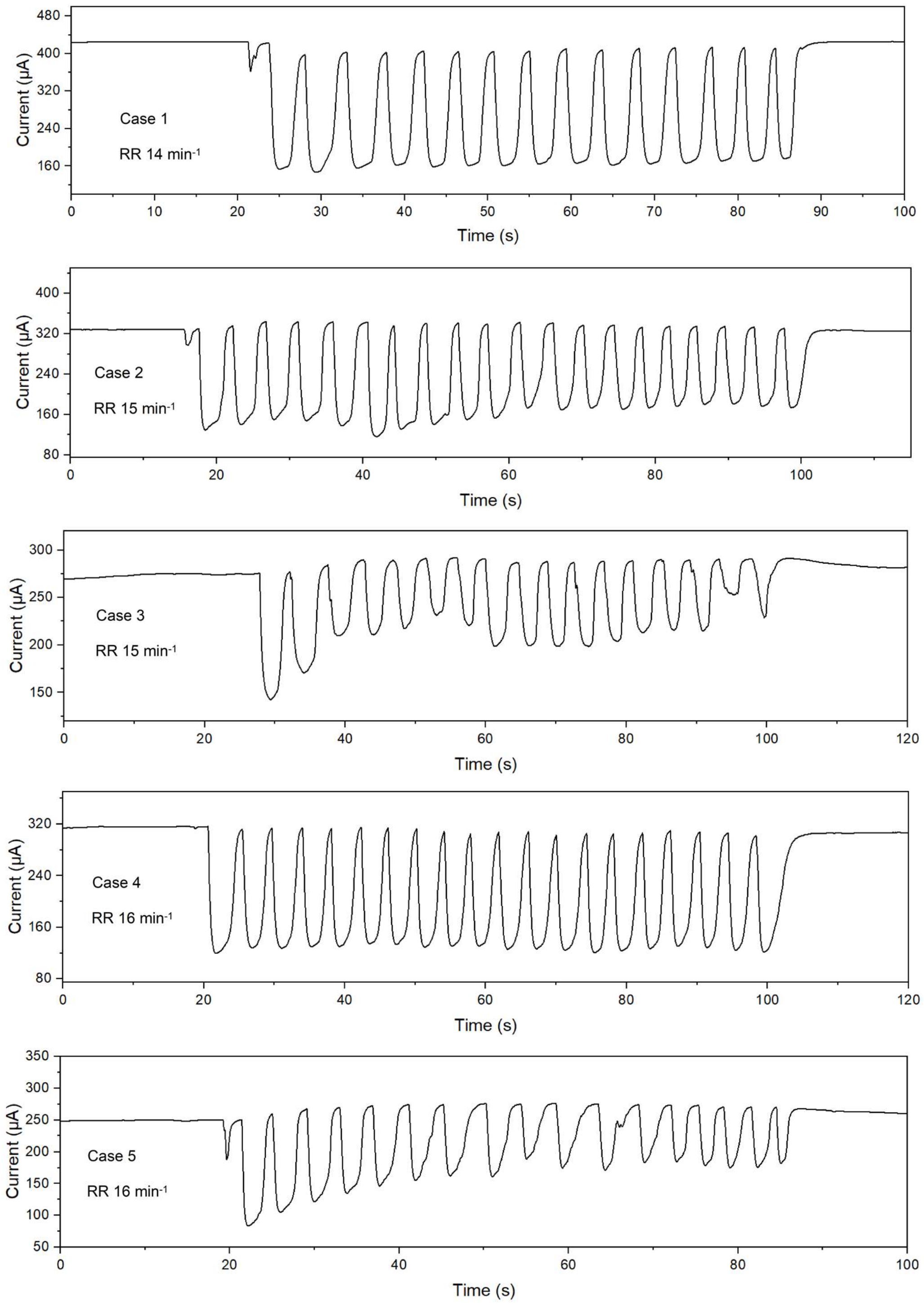

Supplementary Fig. 33| The following breath curves were recorded for the negative case 1 to case 5. Source data are provided as a Source Data file.

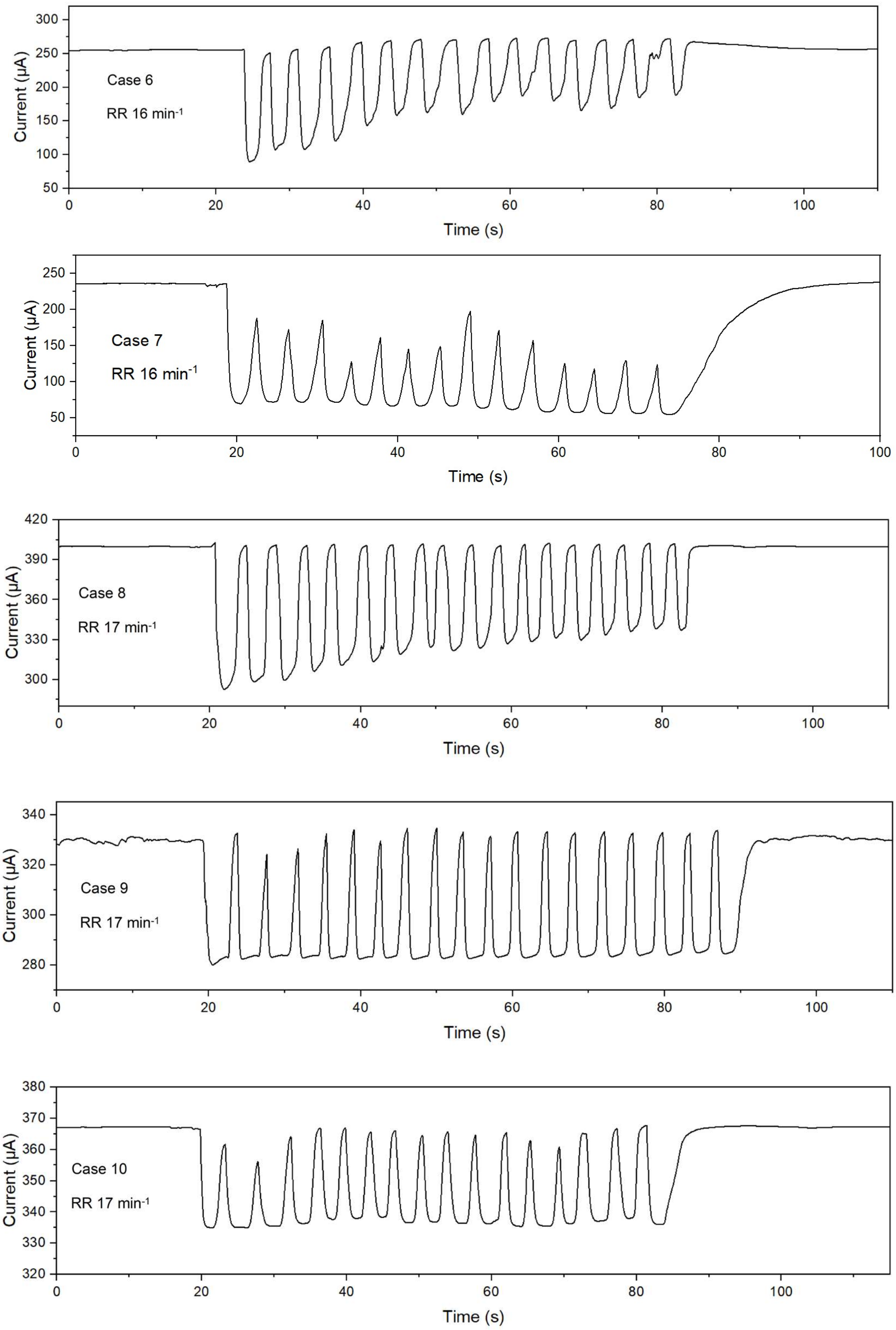

Supplementary Fig. 34| The following breath curves were recorded for negative case 6 to case 10. Source data are provided as a Source Data file.

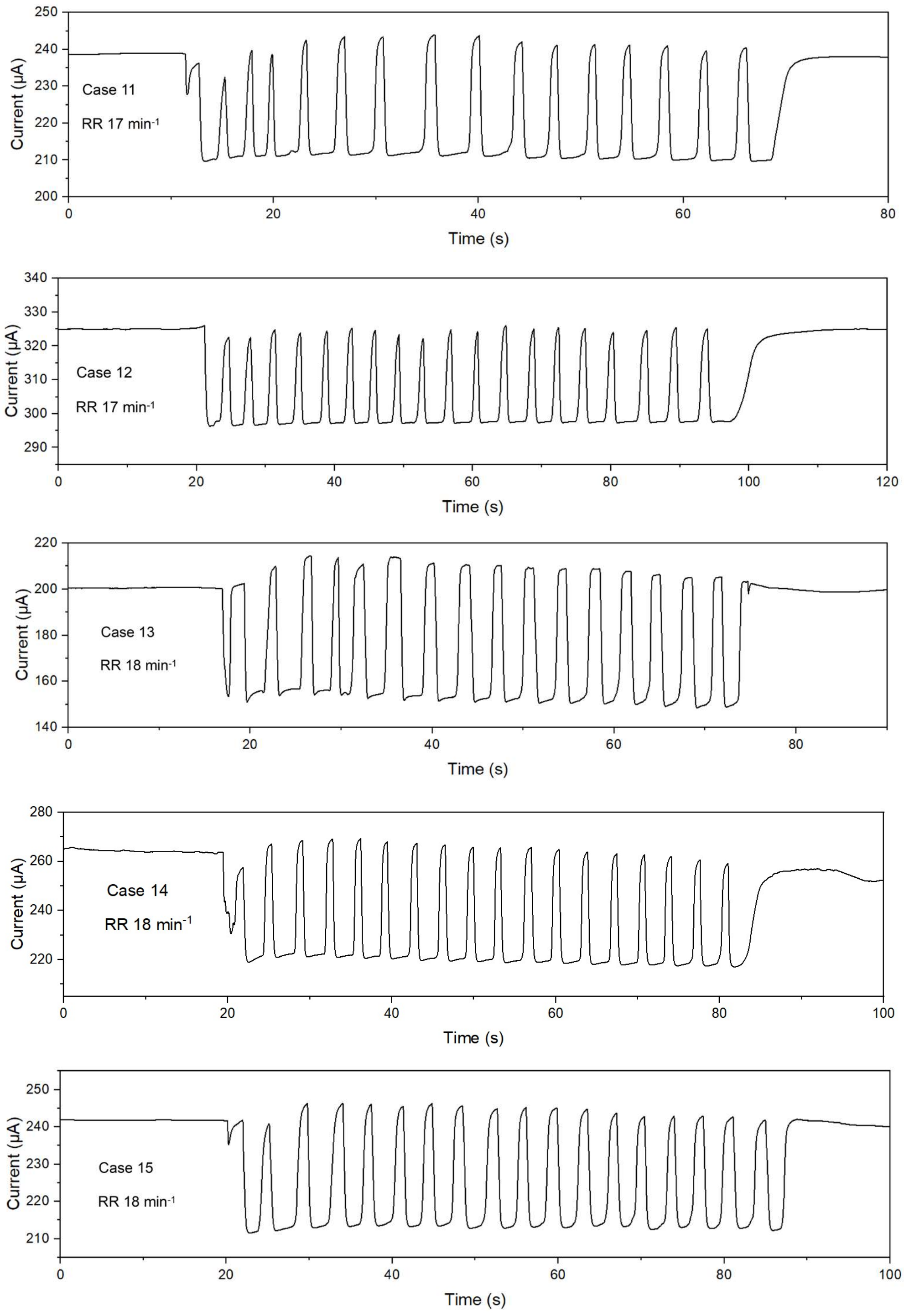

Supplementary Fig. 35| The following breath curves were recorded for negative case 11 to case 15. Source data are provided as a Source Data file.

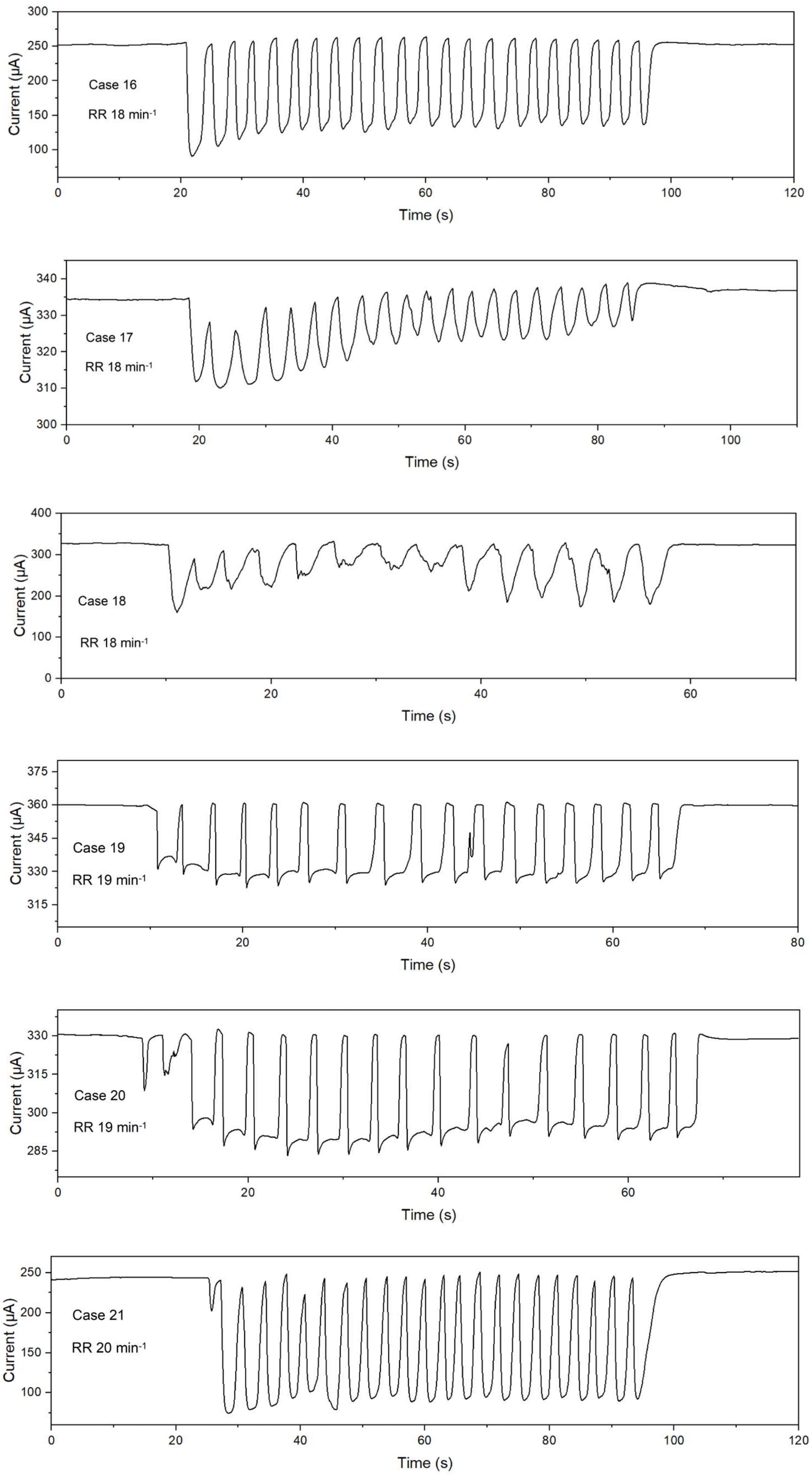

Supplementary Fig. 36| The following breath curves were recorded for negative case 16 to case 21. Source data are provided as a Source Data file.

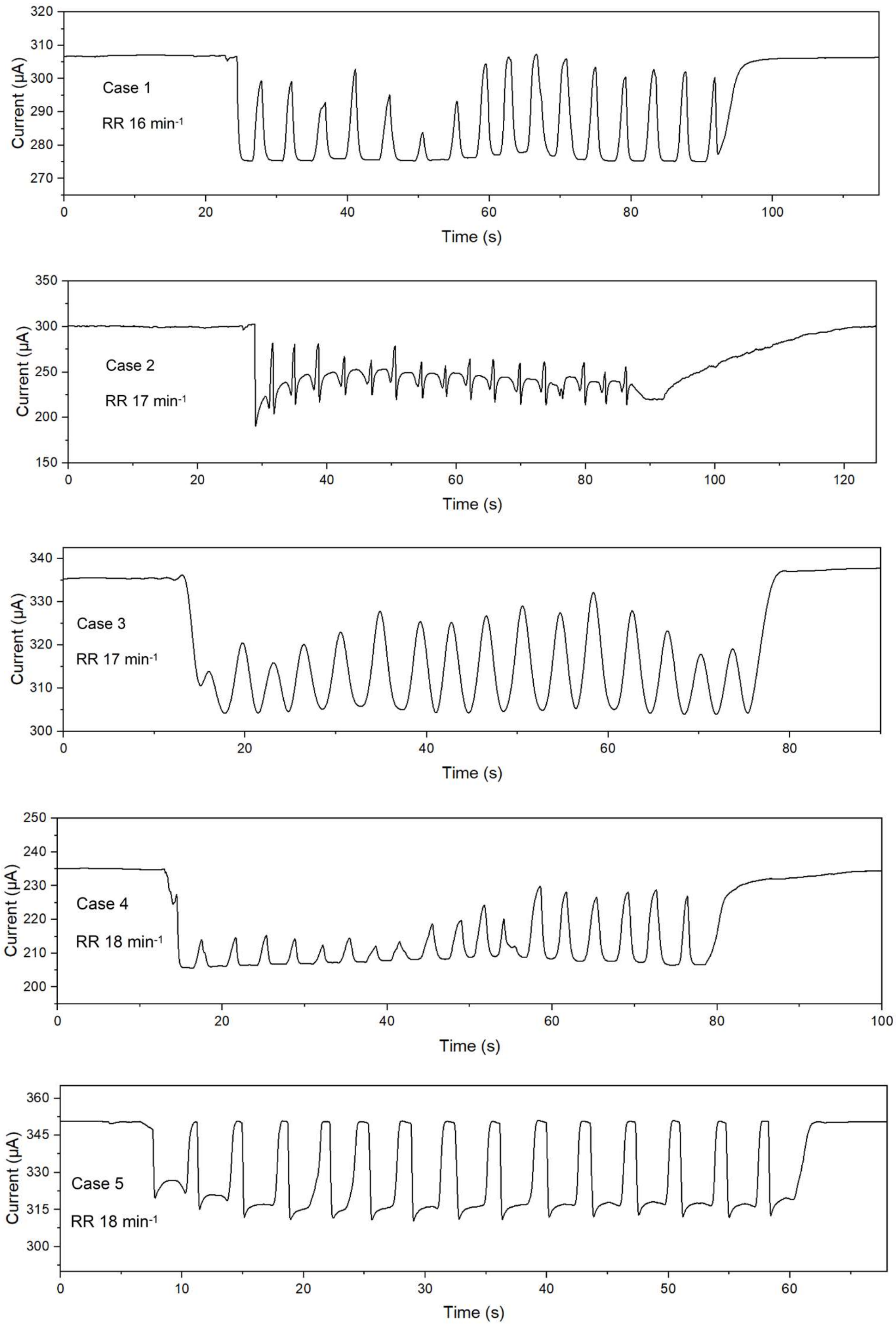

Supplementary Fig. 37| The following breath curves were recorded for the positive case 1 to case 5. Source data are provided as a Source Data file.

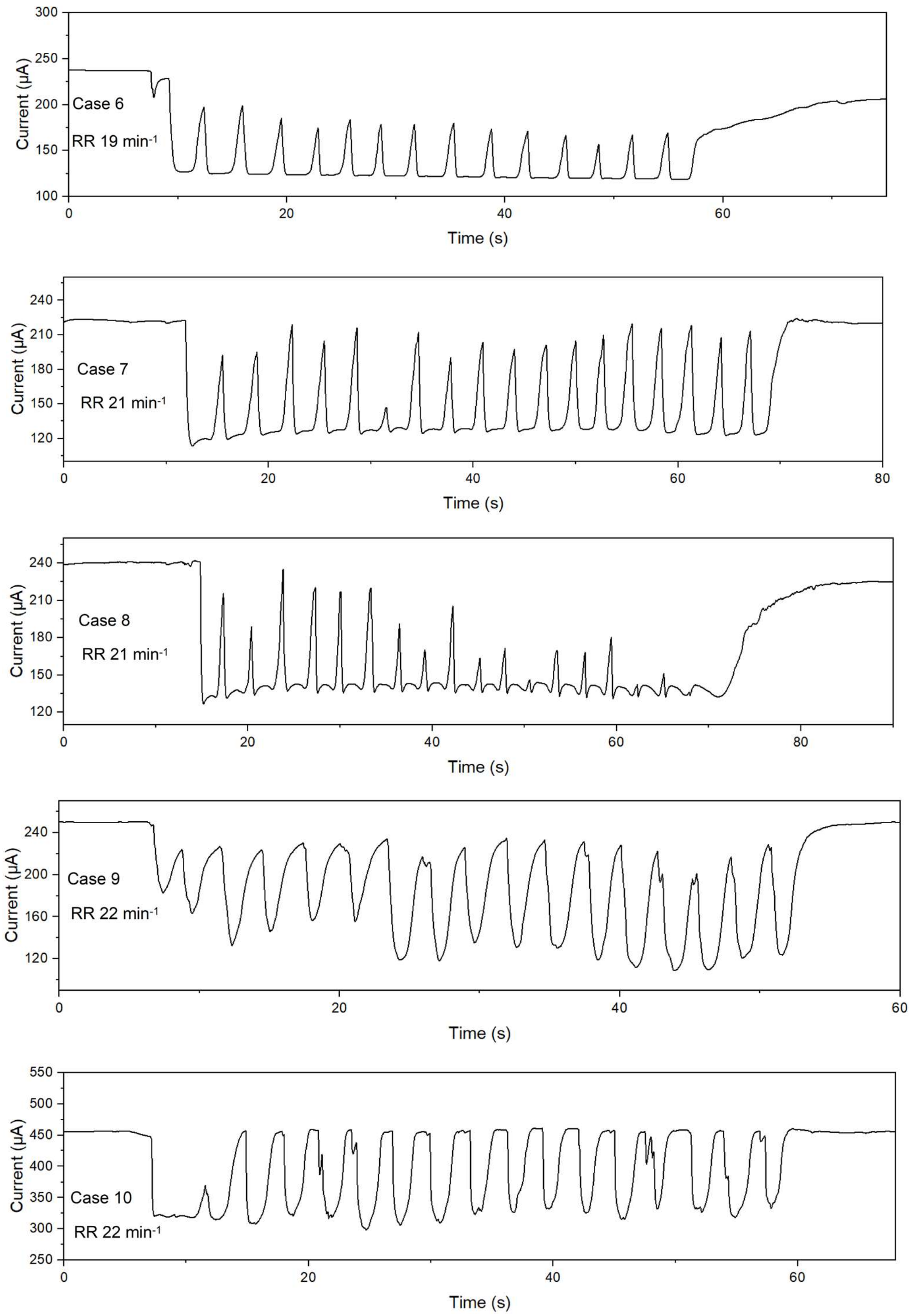

Supplementary Fig. 38| The following breath curves were recorded for the positive case 6 to case 10. Source data are provided as a Source Data file.

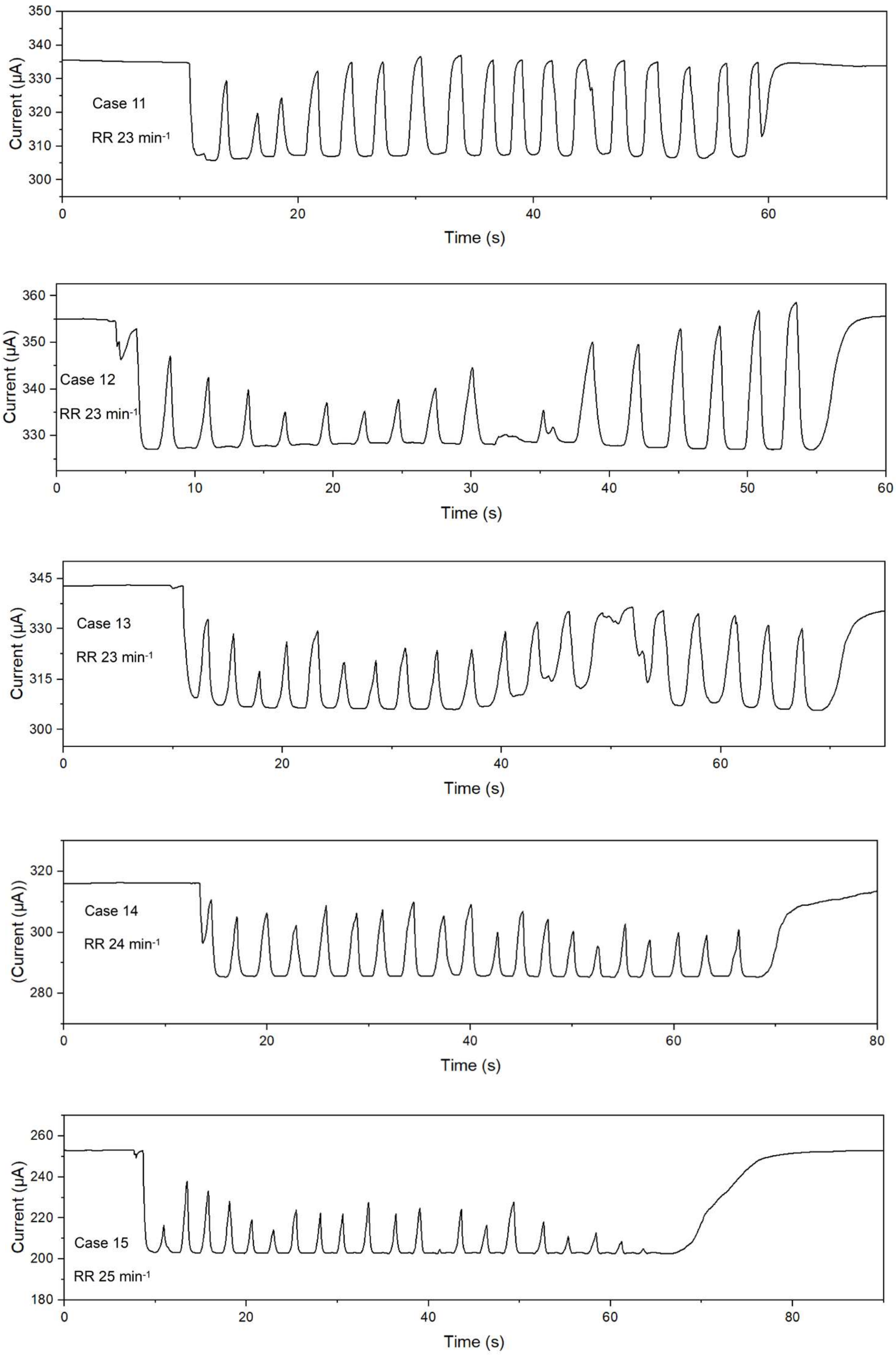

Supplementary Fig. 39| The following breath curves were recorded for the positive case 11 to case 15. Source data are provided as a Source Data file.

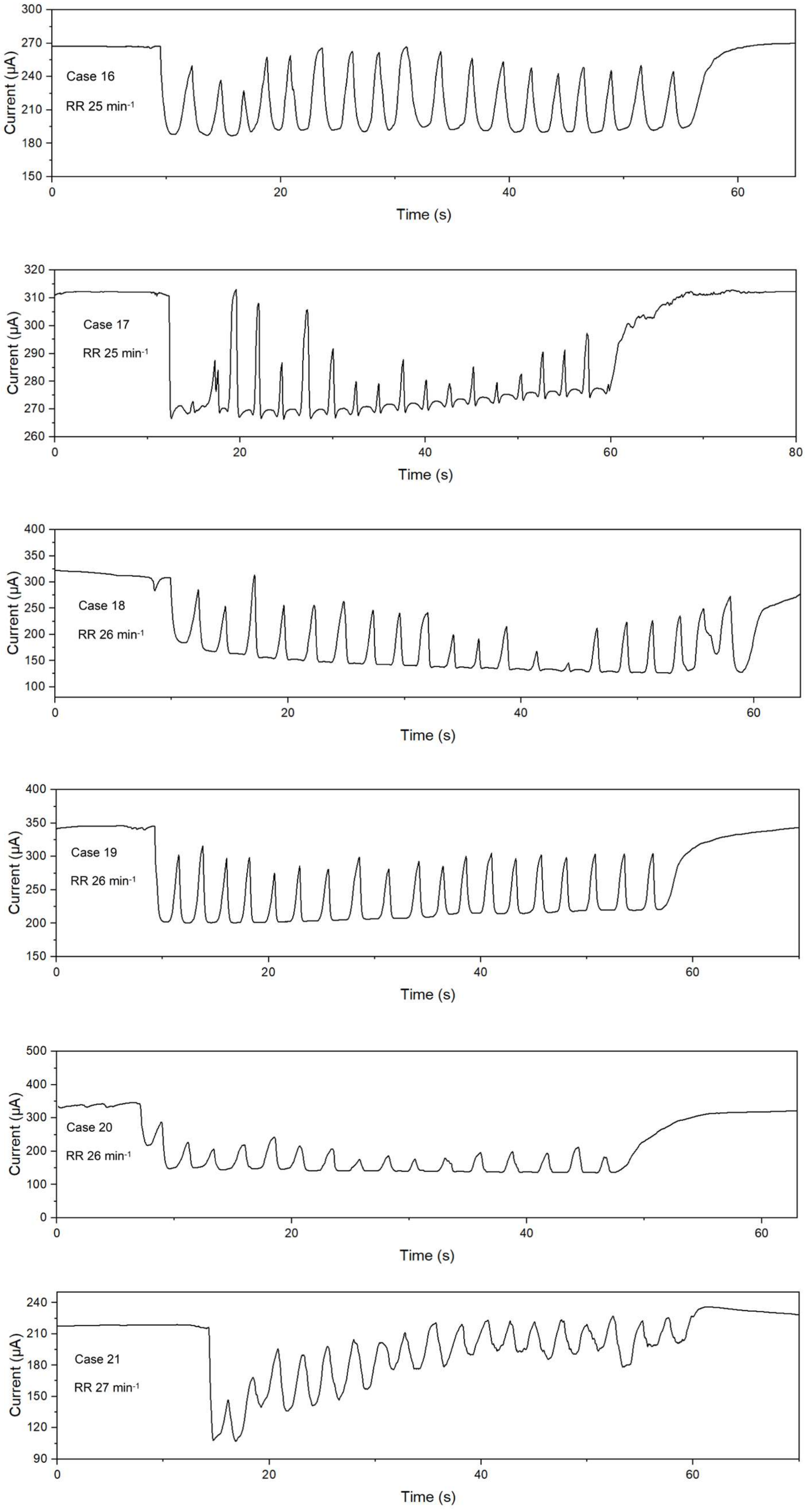

Supplementary Fig. 40| The following breath curves were recorded for the positive case 16 to case 21. Source data are provided as a Source Data file.

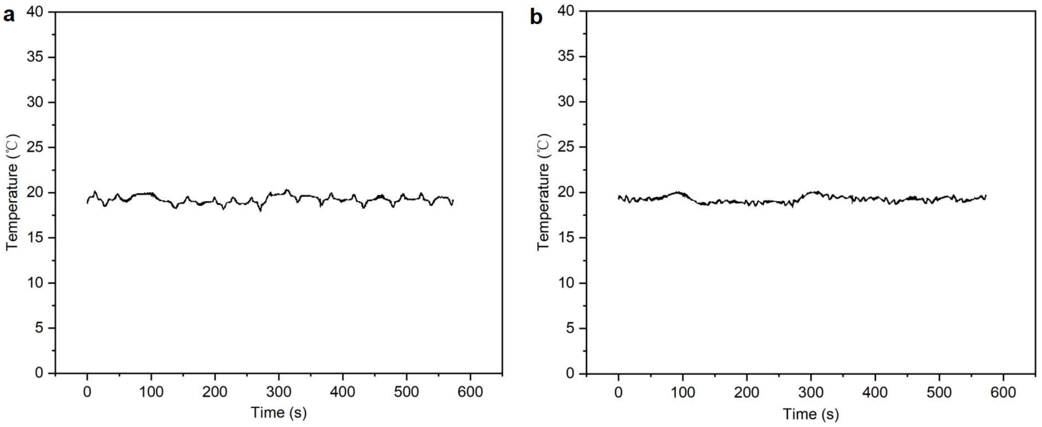

Supplementary Fig. 41| Ambient temperature before and after filtration with the notch filter. **a** Primary ambient temperature curve with low-frequency noise signals (~0.04 Hz). **b** The low-frequency noise signals disappeared after the filtration using the notch filter. Source data are provided as a Source Data file.

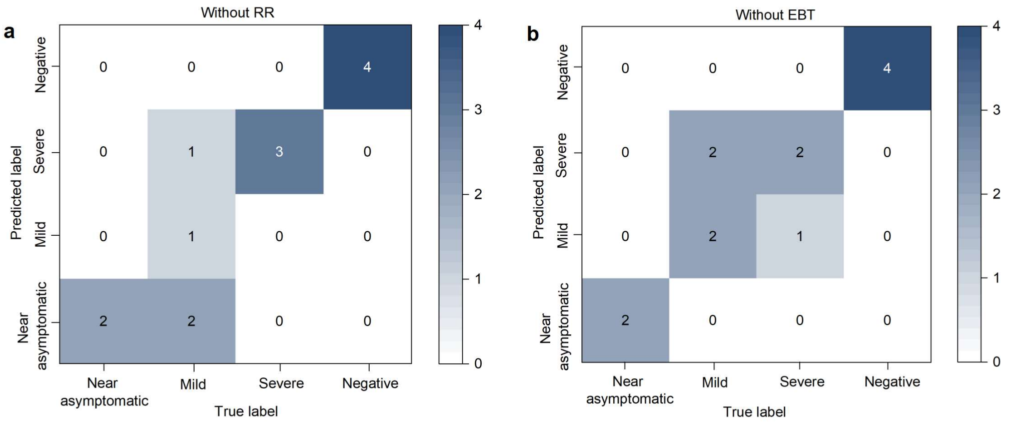

Supplementary Fig. 42| Comparison of accuracy of diagnosing the viral infection without RR and EBT, respectively. a Confusion matrix to predict the positive/negative cases and symptom severity without RR. **b** Confusion matrix to predict the positive/negative cases and symptom severity without EBT.

Considering that RR showed high correlation with EBT (PCC⩾0.7), we verified the influence of adopting both RR and EBT, and only adopting one of them. The diagnosis accuracy without RR was (2+2+1+1+3+4)/13 × 100 % = 100 %. The classification accuracy without RR was (2+1+3+4)/13 × 100 % = 76.9 % (Supplementary Fig. 42a). The diagnosis accuracy without EBT was (2+2+2+2+1+4)/13 × 100 % = 100 %. The classification accuracy without EBT was (2+2+2+4)/13 × 100 % = 76.9 % (Supplementary Fig. 42b). Fig. 5i in the manuscript adopted both RR and EBT. In comparison, the classification accuracy without RR or without EBT was lowered than that of adopting both RR and EBT as features. Therefore, both the RR and EBT should be adopted as features and contribute to classifying the symptom severity. Source data are provided as a Source Data file.

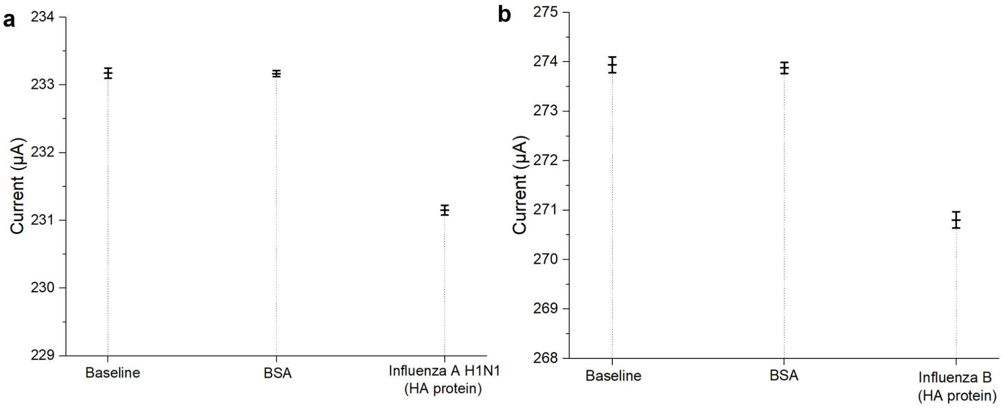

Supplementary Fig. 43| Extension of our technology to another two pandemic viruses. a Specific detection of influenza A H1N1. **b.** Specific detection of influenza B. The test protein concentrations were all 500 fg ml^{-1}. The test temperature was 25 °C. The average current values and error bars were calculated using the recorded 1200 stable current values (n=1200) of each test. Data are presented as mean values ± SD. Source data are provided as a Source Data file.

The Hemagglutinin (HA) antibodies and HA proteins are purchased from Sino Biological. The modification steps are the same as SARS-CoV-2 spike antibodies and spike proteins. The used antigen proteins involved BSA, H1N1 HA protein (Sino Biological, Cat: 11055-V08H) and influenza B HA protein (Sino Biological, Cat: 11053-V08H). BSA was used as the control group. IBS was modified with the influenza A H1N1 HA antibody (Sino Biological; Cat: 11055-MM11; Clone ID: 8F3G7; Dilution: 1 : 4) and influenza B HA antibody (Sino Biological; Cat: 11053-R004; Clone ID: 004; Dilution: 1:4) for specifically detecting influenza A H1N1 HA protein (Cat: 11055-V08H) and influenza B HA protein (Cat: 11053-V08H), respectively. IBS showed no current response to the unreactive BSA protein. IBS showed obvious current response to the H1N1 HA protein and influenza B HA protein. The current change ratios for influenza A H1N1 and influenza B were -0.87 % and -1.1%, respectively. The results indicated that our technology can be also extended to other pandemic virus detection but not limited to the SARS-CoV-2.

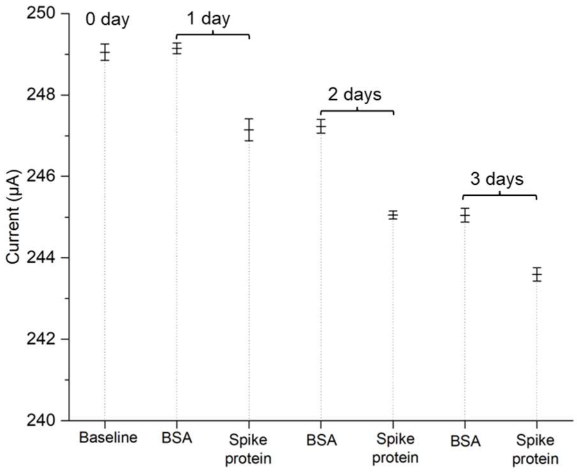

Supplementary Fig. 44| Identification ability of IBS to SARS-CoV-2 spike protein under room temperature. The concentrations of BSA and spike protein were 500 fg ml^{-1}. BSA protein was used for verifying the specificity and the original current value before every test. IBS can still identify the SARS-CoV-2 spike proteins proving the reusable ability during the practical applications. The samples were stored in PBS solution at room temperature (25 ℃) in a sealed petri dish for 3 days. Some water was added in the dish to avoid the PBS evaporation during the storage. The average current values and error bars were calculated using the recorded 1200 stable current values (n=1200) of each test. Data are presented as mean values ± SD. Source data are provided as a Source Data file.

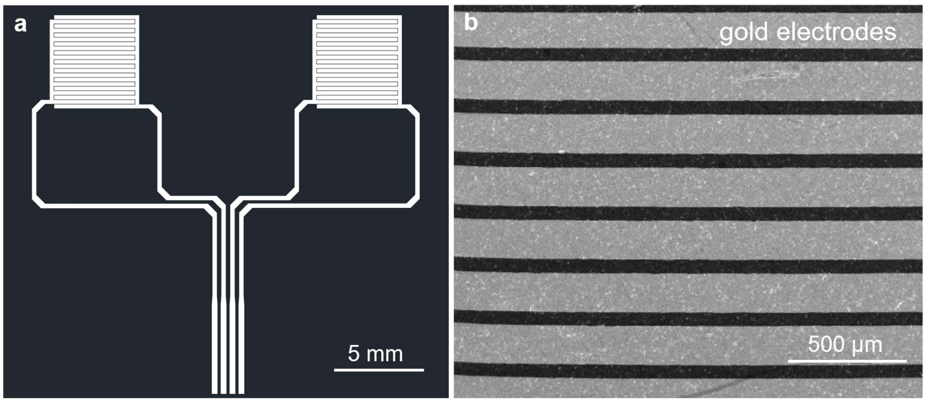

Supplementary Fig. 45| Dimension of the interdigital electrodes. a Structure design of the interdigital electrodes. **b** SEM image of the interdigital electrode.

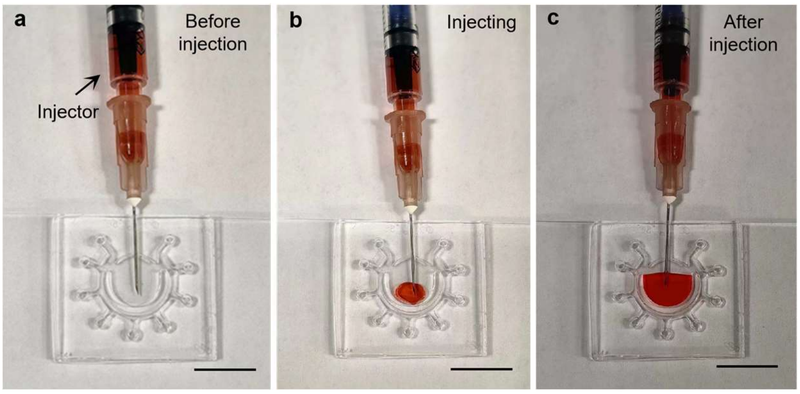

Supplementary Fig. 46| The red ink (or PBS solution) was injected into the chamber from the outlet using an injector. a Picture of the microchannel before injection. **b** Picture of the microchannel with a little red ink in the chamber injected by the injector. **c** Picture of the microchannel with proper amounts of red ink. Scale bar: 1 cm.

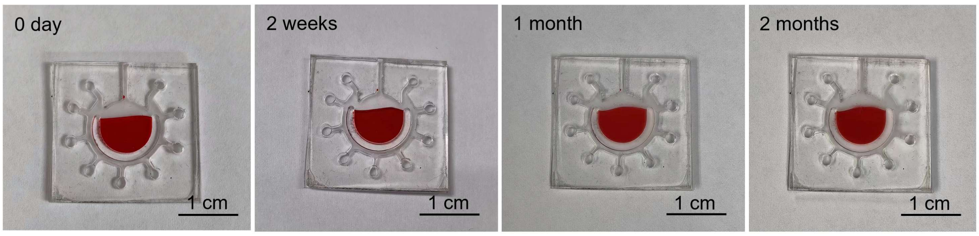

Supplementary Fig. 47| The sample was stored at -20 °C in the refrigerator. There is no observable evaporation and leakage occurred for 2 months.

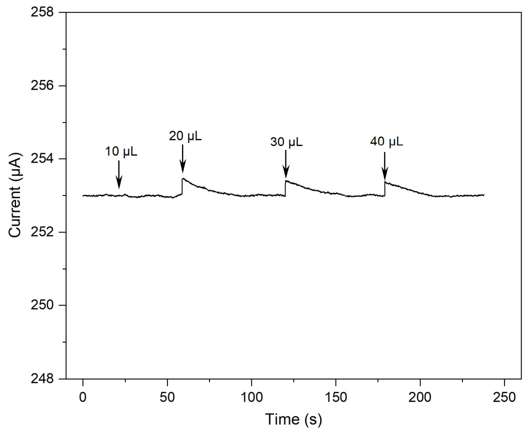

Supplementary Fig. 48| Real-time current curves recorded by dropwise adding 10 μL of PBS on the IBS module. The total volume of 40 μL was added on the biosensor. The current kept stable in different volumes of PBS proving that the liquid volume would not affect the sensitivity of the biosensor. Source data are provided as a Source Data file.

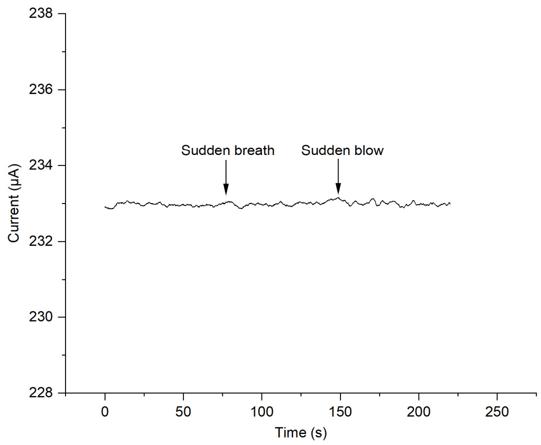

Supplementary Fig. 49| Baseline Current of IBS module when suffered from the sudden breath and blow. The current kept stable whether suffered from a sudden breath or blow, proving the reliability of IBS during the working process, especially under the unknow breath/blow condition. Source data are provided as a Source Data file.

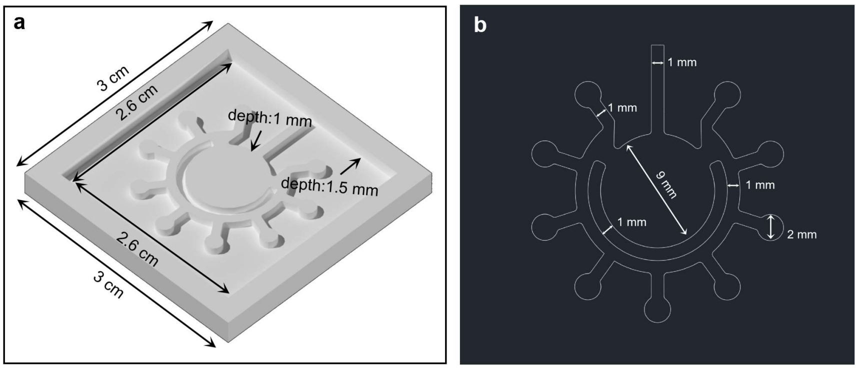

Supplementary Fig. 50| Dimension of the bionic coronavirus inspired microchannel. a Structure diagram of the microchannel mold with size marking. **b** Structure diagram of the two-dimensional microchannel with size marking.

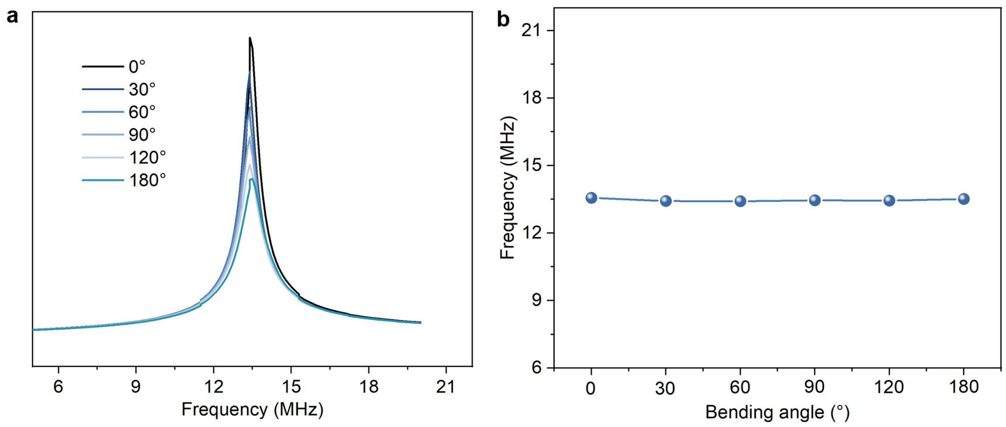

Supplementary Fig. 51| Resonance frequency of the NFC circuit. a Resonance frequency of the NFC circuit at different bending angles from 0° to 180°. **b** The resonance frequency kept stable around 13.56 MHz. Source data are provided as a Source Data file.

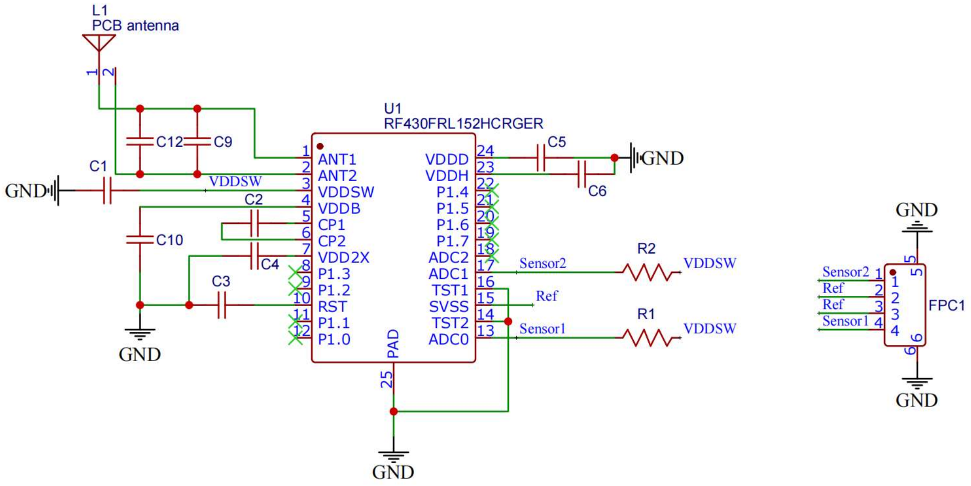

Supplementary Fig. 52| Diagram of the NFC circuit.

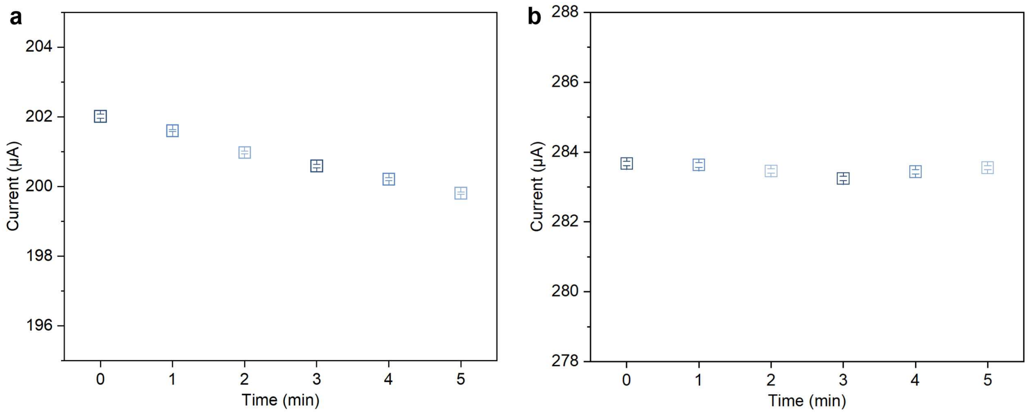

Supplementary Fig. 53| The verification of differentiating exhaled virus from human activities. a Current change of the positive case by blow test. The current change ratio at 5 min was -1.1%. **b** Current change of the negative case by blow test. The current kept stable and showed no decreased tendency. All the tests are carried out when they are eating food and drinking water. The results indicated that the IBS could differentiate the biomarker (i.e., virus) from normal human activities (e.g., food intake). The average current values were calculated using the last 1200 independent data points (n=1200) of each stable current curve. Data are presented as mean values ± SD. Source data are provided as a Source Data file.

Supplementary references:

1. Pan, Y. et al. Viral load of SARS-CoV-2 in clinical samples. Lancet Infect. Dis. **20**, 411-412 (2020).
2. T. Chaibun, et al. Rapid electrochemical detection of coronavirus SARS-CoV-2. Nat. Commun. **12**, 802 (2021).
3. Torres, M. D.T. et al. Low-cost biosensor for rapid detection of SARS-CoV-2 at the point of care. Matter **4**, 2403-2416, 2021.
4. Wang, L. et al. Rapid and ultrasensitive electromechanical detection of ions, biomolecules and SARS-CoV-2 RNA in unamplified samples. Nat. Biomed. Eng. **6**, 276-285 (2022).
5. Puig, H. et al. Minimally instrumented SHERLOCK (miSHERLOCK) for CRISPR-based point-of-care diagnosis of SARS-CoV-2 and emerging variants. Sci. Adv. **7**, eabh2944 (2021).
6. Torrente-Rodríguez, R. M. et al. SARS-CoV-2 RapidPlex: A graphene-based multiplexed telemedicine platform for rapid and low-cost COVID-19 diagnosis and monitoring. Matter **3**, 1981-1998 (2020).
7. Liu, H. et al. Ultrafast, sensitive, and portable detection of COVID-19 IgG using flexible organic electrochemical transistors. Sci. Adv. **7**, eabg8387 (2021).
8. Ganguli, A. et al. Rapid isothermal amplification and portable detection system for SARS-CoV-2. PNAS **117**, 22727-22735 (2020).
9. Xun, G. et al. A rapid, accurate, scalable, and portable testing system for COVID-19 diagnosis. Nat. Commun. **12**, 2905 (2021).
10. Wang, D. et al. Rapid lateral flow immunoassay for the fluorescence detection of SARS-CoV-2 RNA. Nat. Biomed. Eng. **4**, 1150-1158 (2020).
11. Ban, D. K. et al. Rapid self-test of unprocessed viruses of SARS-CoV-2 and its variants in saliva by portable wireless graphene biosensor. Proc. Natl. Acad. Sci. **119**, e2206521119 (2022).
12. Cardozo, K. H. M. et al. Establishing a mass spectrometry-based system for rapid detection of SARS-CoV-2 in large clinical sample cohorts. Nat. Commun. **11**, 6201 (2020).
13. Fozouni, P. et al. Amplification-free detection of SARS-CoV-2 with CRISPR-Cas13a and mobile phone microscopy. Cell **184**, 323-333 (2021).
14. Broughton, J. P. et al. CRISPR-Cas12-based detection of SARS-CoV-2. Nat. Biotech. **38**, 870-874 (2020).

15. Cheong, J. et al. Fast detection of SARS-CoV-2 RNA via the integration of plasmonic thermocycling and fluorescence detection in a portable device. Nat. Biomed. Eng. **4**, 1159-1167 (2020).
16. Pecoraro, V., Negro, A., Pirotti, T. & Trenti, T. Estimate false-negative RT-PCR rates for SARS-CoV-2. A systematic review and meta-analysis. Eur. J. Clin. Invest. **52**, e13706 (2022).
17. World Health Organization, Laboratory testing strategy recommendations for COVID-19, 22 March 2020 (World Health Organization, 2020).
18. Wang, W. et al. Detection of SARS-CoV-2 in different types of clinical specimens. JAMA 323, 1843-1844 (2020).
19. Beeching, N. J., Fletcher, T. E. & Beadsworth, M. B. J. Covid-19: testing times. BMJ **369**,1403 (2020).
20. Giovanninim, G., Haick, H. & Haick, H. Detecting COVID-19 from breath: A game changer for a big challenge. ACS Sens. **6**, 1408−1417 (2021).
21. Wang, C. C. et al. Airborne transmission of respiratory viruses. Science **373**, eabd9149 (2021).
22. Prather, K. A. C., Wang, C. & Schooley, R. T. Reducing transmission of SARS-CoV-2. Science **368**, 1422-1424 (2020).
23. Leung, N. H. L. et al. Respiratory virus shedding in exhaled breath and efficacy of face masks. Nat. Med. **26**, 676−680 (2020).
24. Gould, O., Ratcliffe, N., Krol, E. & Costello, B. L. Breath analysis for detection of viral infection, the current position of the field. J. Breath Res. **14**, 041001 (2020).
25. Ates, H. C. & Dincer, C. Wearable breath analysis. Nat. Rev. Bioeng. **1**, 80-82 (2023).
26. Wang, B. et al. Wearable bioelectronic masks for wireless detection of respiratory infectious diseases by gaseous media. Matter **5**, 1-16 (2022).
27. Nguyen, P. Q. et al. Wearable materials with embedded synthetic biology sensors for biomolecule detection. Nat. Biotech. **39**, 1366-1374 (2021).
