# Supplementary material for: Wireless, battery-free, multifunctional integrated bioelectronics for respiratory pathogens monitoring and severity evaluation
Source: Nat Commun. 2023 Nov 20;14:7539. doi: 10.1038/s41467-023-43189-z (PMC10661182; doi:10.1038/s41467-023-43189-z)
Supplement: Supplementary file 3 — Description of Additional Supplementary Files [file 41467_2023_43189_MOESM3_ESM.docx]

**Description of Additional Supplementary Files**

**Supplementary Movies**

**Supplementary Movie 1.** Rapid passive screening of positive cases in public gathering places with the assistance of radio-frequency biosafety doors. PIDS was attached on the inner surface of a face mask. When the positive individuals walked through the door, it emitted red light indicating virus infection with need of further isolation.

**Supplementary Movie 2.** Rapid passive screening of negative cases in public gathering places with the assistance of radio-frequency biosafety doors. PIDS was attached on the inner surface of a face mask. When the healthy negative individuals walked through the door, it emitted green light indicating the safe health status.

**Supplementary Movie 3.** Active self-diagnosis of negative cases by breath analysis. The PIDS was attached on the inner surface of a face mask. The user can rapidly conduct the self-diagnosis just by the smartphone touch. The PIDS rapidly and accurately diagnosed the health status and severity as “negative” and “safe”.

**Supplementary Movie 4.** Active self-diagnosis of asymptomatic positive cases by breath analysis. The PIDS was attached on the inner surface of a face mask. The user can rapidly conduct the self-diagnosis just by the smartphone touch. The PIDS rapidly and accurately diagnosed the infection status and severity as “positive” and “asymptomatic”.
