# Supplementary material for: Wireless, battery-free, multifunctional integrated bioelectronics for respiratory pathogens monitoring and severity evaluation
Source: Nat Commun. 2023 Nov 20;14:7539. doi: 10.1038/s41467-023-43189-z (PMC10661182; doi:10.1038/s41467-023-43189-z)
Supplement: Supplementary file 8 — Reporting Summary [file 41467_2023_43189_MOESM8_ESM.pdf]

nature portfolio

Corresponding author(s): Jiandong Huang, Lingqian Chang, Xinge Yu

Last updated by author(s): Oct 1, 2023

Reporting Summary

Nature Portfolio wishes to improve the reproducibility of the work that we publish. This form provides structure for consistency and transparency in reporting. For further information on Nature Portfolio policies, see our Editorial Policies and the Editorial Policy Checklist.

Statistics

For all statistical analyses, confirm that the following items are present in the figure legend, table legend, main text, or Methods section.

n/a	Confirmed	
☐	☒	The exact sample size (*n*) for each experimental group/condition, given as a discrete number and unit of measurement
☐	☒	A statement on whether measurements were taken from distinct samples or whether the same sample was measured repeatedly
☒	☐	The statistical test(s) used AND whether they are one- or two-sided<br>*Only common tests should be described solely by name; describe more complex techniques in the Methods section.*
☒	☐	A description of all covariates tested
☒	☐	A description of any assumptions or corrections, such as tests of normality and adjustment for multiple comparisons
☐	☒	A full description of the statistical parameters including central tendency (e.g. means) or other basic estimates (e.g. regression coefficient) AND variation (e.g. standard deviation) or associated estimates of uncertainty (e.g. confidence intervals)
☒	☐	For null hypothesis testing, the test statistic (e.g. *F*, *t*, *r*) with confidence intervals, effect sizes, degrees of freedom and *P* value noted<br>*Give P values as exact values whenever suitable.*
☒	☐	For Bayesian analysis, information on the choice of priors and Markov chain Monte Carlo settings
☒	☐	For hierarchical and complex designs, identification of the appropriate level for tests and full reporting of outcomes
☒	☐	Estimates of effect sizes (e.g. Cohen's *d*, Pearson's *r*), indicating how they were calculated

Our web collection on statistics for biologists contains articles on many of the points above.

Software and code

Policy information about availability of computer code

Data collection	atomic force microscope (AFM, Asylum Research, MFP-3D; NanoScope TappingMode in Air);<br>scanning electron microscope (SEM, Quanta 450 FEG; xT microscope Control v6.2.4 build 3069);<br>energy dispersive X-ray (EDX; IXRF SYSTEMS, Model 550i);<br>X-ray photoelectron spectroscopy (XPS, Thermo Scientific K-Alpha);<br>contact angle meter (Dataphysics OCA20);<br>electrochemical workstation (CHI660E);<br>Keysight B1500A Semiconductor Analyzer (Keysight EasyEXPERT Software);<br>HIOKI LR8431-30 MEMORY HiLOGGER;
Data analysis	AutoCAD 2023;<br>Origin 2021;<br>NanoScope Analysis 1.7;<br>COMSOL Multiphysics 5.5;<br>Corel VideoStudio 2021;<br>Python 3.9.13;

For manuscripts utilizing custom algorithms or software that are central to the research but not yet described in published literature, software must be made available to editors and reviewers. We strongly encourage code deposition in a community repository (e.g. GitHub). See the Nature Portfolio guidelines for submitting code & software for further information.

Data

Policy information about availability of data

All manuscripts must include a data availability statement. This statement should provide the following information, where applicable:

- Accession codes, unique identifiers, or web links for publicly available datasets
- A description of any restrictions on data availability
- For clinical datasets or third party data, please ensure that the statement adheres to our policy

Data availability: All data supporting the findings described in this manuscript are available in the article and the Supplementary Information. Source data are provided with this paper. Code availability: The machine learning data used in this study are available in the Github under accession code [https://github.com/veracoding/Demo.git].

Research involving human participants, their data, or biological material

Policy information about studies with human participants or human data. See also policy information about sex, gender (identity/presentation), and sexual orientation and race, ethnicity and racism.

Reporting on sex and gender	We randomly recruited 42 volunteers in this experiment, including 21 positive cases and 21 negative cases. The positive cases include 10 males and 11 females. The negative cases include 10 males and 11 females. The gender showed no influence on SARS-CoV-2 infection. For the positive cases, three of them live in a family. Eight of them are couples. The others are independent human subjects.
Reporting on race, ethnicity, or other socially relevant groupings	They are all Chinese Han populations. Some of them are Hong Kong residents. The others came from the mainland. We have not recorded the detailed numbers of the Hong Kong residents and people from the mainland. Our test only contains the breath and blow, no other biological materials are collected.
Population characteristics	We showed the details of the participants in Table 3 and Table 4. The ages of the positive cases range between 20 and 59. The ages of negative cases are between 19 and 31. For the positive cases, they are infected for the first time. They are taking the medication to fight the virus when we carry out the experiment. For the negative cases, five of them have been infected and recovered health. For the 42 participants, two cases have not been vaccinated before the experiment. The others have been vaccinated before they are infected.
Recruitment	We randomly recruited the positive participants in society. Most of the infected participants were introduced by friends when they were self-isolated at home. The others were recruited by the recruitment advertisement. All of them were confirmed as infection by rapid detection kit or PCR in hospitals by themselves. When we conducted the test, we further confirmed their infection status by rapid detection kits and provided them with informed consents. The negative participants were randomly recruited in campus by the recruitment advertisement. For all the participants, we have no potential self-selection bias. When they participated the experiment, we further confirmed their health status using rapid detection kits and provided them with informed consents.
Ethics oversight	All participants have provided informed consent when they took part in this experiment. All human experiments were performed in accordance with protocols approved by the Institutional Review Board of the University of Hong Kong/Hospital Authority Hong Kong West Cluster (UW 23-107). The authors affirm that human research participants provided written informed consent for the publication of the images in Figure 1g, Supplementary Figure 2a-c, Supplementary Table 2.

Note that full information on the approval of the study protocol must also be provided in the manuscript.

Field-specific reporting

Please select the one below that is the best fit for your research. If you are not sure, read the appropriate sections before making your selection.

☒ Life sciences ☐ Behavioural & social sciences ☐ Ecological, evolutionary & environmental sciences

For a reference copy of the document with all sections, see nature.com/documents/nr-reporting-summary-flat.pdf

Life sciences study design

All studies must disclose on these points even when the disclosure is negative.

Sample size	(1) 42 volunteers have been recruited in this experiment. The sample size is comparable with the reported studies for COVID-19 detection [1-16].<br>(2) We have not performed the sample size calculation. We first tested the device performance on spike protein (eight concentrations were tested), pseudo virus (nine concentrations were tested) and live virus (six concentrations were tested) in turn. Twelve devices were used in these experiments with good reproducibility. Then we further verified the device performance on human. For the breath test of each participant, we measured seven curves at different time (0 min, 5 min, 10 min, 15 min, 20 min, 25 min, 30 min). 294 tests in total were performed for 42 participants. For the blow test of each participant, we measured six curves at different time (0 min, 1 min, 2 min, 3 min, 4 min, 5 min). 252 tests in total were performed for 42 participants. These test sample sizes were much larger than that in literatures [1-16], which were sufficient in this study. 84 devices were tested for the breath and blow test, which was sufficient and enough to evaluate the device performance.

(3) For machine learning, we selected data of 29 volunteers for training, the sample size of remained 13 volunteers (25% of total sample size) was sufficient for prediction of virus infection and severity evaluation.
(4) In our experiment, we need to calculate the threshold value of stable current (or current change ratio) to redefine the positive case and negative case, which fluctuated within a narrow range. So it reasonable to calculate the average current value of each single device. Our device showed stable current curve after 50s. We tested the curve for 5 min to ensure the best stability. In our experiment, we calculated the average current value and error bar within 1 minute at the 5th min of each single device. The sample size for average current value and error bar was calculated by n = 1200 data points. Sample frequency was 20 points per second. This method can be used to reflect the fluctuation range of stable current and evaluate the current drift of each device on each test curve. The comparison of all the data of current change ratio calculated from all devices (n=84) can be used to form the threshold value (-0.2%) to differentiate the positive from the negative. n = 210 for blow test. n=252 for breath test. The sample size was sufficient to set the standard of our device to diagnose the positive and negative subjects.
References:
1. Chaibun, T. et al. Rapid electrochemical detection of coronavirus SARS-CoV-2. Nat. Commun. 12, 802 (2021).
2. Torres, M. D. T., Araujo, W. R., Lima, L. F., Ferreira, A. L. & Fuente-Nunez, C. Low-cost biosensor for rapid detection of SARS-CoV-2 at the point of care. Matter 4, 2403-2416 (2021).
3. Wang, L. et al. Rapid and ultrasensitive electromechanical detection of ions, biomolecules and SARS-CoV-2 RNA in unamplified samples. Nat. Biomed. Eng. 6, 276-285 (2022).
4. Puig, H. et al. Minimally instrumented SHERLOCK (miSHERLOCK) for CRISPR-based point-of-care diagnosis of SARS-CoV-2 and emerging variants. Sci. Adv. 7, eabh2944 (2021).
5. Torrente-Rodríguez, R. M. et al. SARS-CoV-2 RapidPlex: A graphene-based multiplexed telemedicine platform for rapid and low-cost COVID-19 diagnosis and monitoring. Matter 3, 1981-1998 (2020).
6. Liu, H. et al. Ultrafast, sensitive, and portable detection of COVID-19 IgG using flexible organic electrochemical transistors. Sci. Adv. 7, eabg8387 (2021).
7. Ganguli, A. et al. Rapid isothermal amplification and portable detection system for SARS-CoV-2. Proc. Natl. Acad. Sci. 117, 22727-22735 (2020).
8. Xun, G., Lane, S. T., Petrov, V. A., Pepa, B. E. & Zhao, H. A rapid, accurate, scalable, and portable testing system for COVID-19 diagnosis. Nat. Commun. 12, 2905 (2021).
9. Wang, D. et al. Rapid lateral flow immunoassay for the fluorescence detection of SARS-CoV-2 RNA. Nat. Biomed. Eng. 4, 1150-1158 (2020).
10. Guo, K. et al. Rapid single-molecule detection of COVID-19 and MERS antigens via nanobody-functionalized organic electrochemical transistors. Nat. Biomed. Eng. 5, 666-677 (2021).
11. Ban, D. K. et al. Rapid self-test of unprocessed viruses of SARS-CoV-2 and its variants in saliva by portable wireless graphene biosensor. Proc. Natl. Acad. Sci. 119, e2206521119 (2022).
12. Seo, G. et al. Rapid detection of COVID-19 causative virus (SARS-CoV-2) in human nasopharyngeal swab specimens using field-effect transistor based biosensor. ACS Nano 14, 5135-5142 (2020).
13. Cardozo, K. H. M. et al. Establishing a mass spectrometry-based system for rapid detection of SARS-CoV-2 in large clinical sample cohorts. Nat. Commun. 11, 6201 (2020).
14. Fozouni, P. et al. Amplification-free detection of SARS-CoV-2 with CRISPR-Cas13a and mobile phone microscopy. Cell 184, 323-333 (2021).
15. Broughton, J. P. et al. CRISPR-Cas12-based detection of SARS-CoV-2. Nat. Biotech. 38, 870-874 (2020).
16. Cheong, J. et al. Fast detection of SARS-CoV-2 RNA via the integration of plasmonic thermocycling and fluorescence detection in a portable device. Nat. Biomed. Eng. 4, 1159-1167 (2020).

Data exclusions

No data point was excluded.

Replication

All experiments were replicated at least once and the experimental results were repeatable.

Randomization

(1) Before the test, we first confirm the health status of participants using rapid detection kit. The infected patients are allocated into positive group. The uninfected health participants are allocated into negative group. Therefore, the group allocation is not random. But the case selection for machine learning prediction is random to verify the prediction ability of our model in evaluating the infection status and symptom severity. (2) After we collected all the current variation data, we compared the data of positive cases with those of negative cases, we used the threshold value -0.2% to redefine the positive and negative participants. When the current variation value was lower than -0.2%, it was defined as "positive". When the current variation value was higher than -0.2%, it was defined as "negative". So, the allocation of positive group and negative group here was also not random. The threshold value was the only standard with no covariates. (3) When classify the symptom severity group, fever was selected as a standard to differentiate the "severe" from "mild". The fever was the only standard with no covariates. The secondary symptoms (e.g., sore throat, cough, sore muscle) were used as standards to differentiate the "mild" from near "asymptomatic". The secondary symptoms have a certain randomness, which are self-reported by participants themselves. (4) The other remained experiments do not involve allocations.

Blinding

To obtain the evaluation standard of our created device to differentiate the positive and the negative, we need to know the health status of participants before the tests and compare the current variation data of positive and negative participants. Then we obtain the threshold value (-0.2%) of current variation, which was used to redefine the positive and negative status. To ensure the infection status and eliminate the false health status, each subject was verified using the commercial rapid test kit before the test. At this point, the positive status and negative status were classified using the commercial rapid test kit. Therefore, the data collection for positive volunteers and negative volunteers are not blinded. The collected data were used to redefine the positive status and negative status by the threshold value (-0.2%) of current change ratio of our device. According to the threshold value, the diagnosis accuracy can be calculated at different breath time and blow time. After that, the data of 29 volunteers (not blinded) were used for machine learning training, the data of remained 13 volunteers are used for testing the prediction ability of infection status and symptom severity. At this point, the prediction test is a blind test.

Reporting for specific materials, systems and methods

We require information from authors about some types of materials, experimental systems and methods used in many studies. Here, indicate whether each material, system or method listed is relevant to your study. If you are not sure if a list item applies to your research, read the appropriate section before selecting a response.

Materials & experimental systems

n/a	Involved in the study	
☐	☒	Antibodies
☐	☒	Eukaryotic cell lines
☒	☐	Palaeontology and archaeology
☒	☐	Animals and other organisms
☒	☐	Clinical data
☒	☐	Dual use research of concern
☒	☐	Plants

Methods

n/a	Involved in the study	
☒	☐	ChIP-seq
☒	☐	Flow cytometry
☒	☐	MRI-based neuroimaging

Antibodies

Antibodies used	SARS-CoV-2 spike antibody 1 (Sino Biological; Cat: 40150-R007; Clone ID: 007; Dilution, 1:4), SARS-CoV-2 spike antibody 2 (Sino Biological; Cat:40592-MM57; Clone ID: 57; Dilution, 1:4) and SARS-CoV-2 spike antibody 3 (Sino Biological; Cat: 40591-MM48; Clone ID: 48; Dilution: 1:4). Influenza A H1N1 HA antibody (Sino Biological; Cat: 11055-MM11; Clone ID: 8F3G7; Dilution: 1:4) and influenza B HA antibody (Sino Biological; Cat: 11053-R004; Clone ID: 004; Dilution: 1:4).
Validation	https://cn.sinobiological.com/antibodies/cov-spike-40150-r007;<br>https://cn.sinobiological.com/antibodies/cov-spike-40592-mm57;<br>https://www.sinobiological.com/antibodies/cov-spike-40591-mm48;<br>https://cn.sinobiological.com/antibodies/hemagglutinin-ha-11055-mm11;<br>https://cn.sinobiological.com/antibodies/hemagglutinin-ha-11053-r004;

Eukaryotic cell lines

Policy information about cell lines and Sex and Gender in Research

Cell line source(s)	HEK293T (ATCC, Cat # CRL-3216)and Vero E6(ATCC, Cat # CRL-1586) cell lines were purchased from ATCC (Manassas, VA, USA). Vero E6 was transfected with TMPRSS2 plasmid (Sino Biological, Cat # HG13070-CH) for further use.
Authentication	Certificate of analysis of the cells lines used came along with the purchase from the merchants listed above. Short Tandem Repeat (STR) profiling is used for above cell lines' authentication.
Mycoplasma contamination	All cell lines were regularly tested for mycoplasma contamination (Mycoplasma Detection Kit, SouthernBiotech, 13100-01) and were negative.
Commonly misidentified lines<br>(See ICLAC register)	No cell lines listed as commonly misidentified were used in this study.
